# Outer Membrane Proteins form Specific Patterns in Antibiotic-Resistant *Edwardsiella tarda*

**DOI:** 10.3389/fmicb.2017.00069

**Published:** 2017-02-02

**Authors:** Bo Peng, Chao Wang, Hui Li, Yu-bin Su, Jin-zhou Ye, Man-jun Yang, Ming Jiang, Xuan-xian Peng

**Affiliations:** Center for Proteomics and Metabolomics, State Key Laboratory of Bio-Control, Guangdong Province Key Laboratory for Pharmaceutical Functional Genes, School of Life Sciences, Sun Yat-sen UniversityGuangzhou, China

**Keywords:** outer membrane proteins, outer membrane protein pattern, antibiotics, antibiotic resistance, *Edwardsiella tarda*

## Abstract

Outer membrane proteins of Gram-negative bacteria play key roles in antibiotic resistance. However, it is unknown whether outer membrane proteins that respond to antibiotics behave in a specific manner. The present study specifically investigated the differentially expressed outer membrane proteins of an antibiotic-resistant bacterium, *Edwardsiella tarda*, a Gram-negative pathogen that can lead to unnecessary mass medication of antimicrobials and consequently resistance development in aquaculture and a spectrum of intestinal and extraintestinal diseases in humans. The comparison of a clinically isolated strain to the laboratory derived kanamycin-, tetracycline-, or chloramphenicol-resistant strains identified their respective outer membrane proteins expression patterns, which are distinct to each other. Similarly, the same approach was utilized to profile the patterns in double antibiotic-resistant bacteria. Surprisingly, one pattern is always dominant over the other as to these three antibiotics; the pattern of chloramphenicol is over tetracycline, which is over kanamycin. This type of pattern was also confirmed in clinically relevant multidrug-resistant bacteria. In addition, the presence of plasmid encoding antibiotic-resistant genes also alters the outer membrane protein profile in a similar manner. Our results demonstrate that bacteria adapt the antibiotic stress through the regulation of outer membrane proteins expression. And more importantly, different outer membrane protein profiles were required to cope with different antibiotics. This type of specific pattern provides the rationale for the development of novel strategy to design outer membrane protein arrays to identify diverse multidrug resistance profiles as biomarkers for clinical medication.

## Introduction

The emergence of antibiotic-resistant bacteria is a major threat to public health and animal farming (Lee and Collins, 2012; Brynildsen et al., 2013; Lewis, 2013; Peng et al., 2015). Antibiotic resistance can generally be classified as intrinsic resistance and acquired resistance. Acquired resistance is insusceptible to a particular antibiotic in a bacterial strain by means of mutation in its own DNA or acquisition of resistance-conferring DNA from another source, while intrinsic resistance is the innate ability of a bacterial species to resist an antibiotic class through its inherent structural or functional characteristics (Li et al., 2015). In Gram-negative bacteria, intrinsic resistance is associated with the regulation of outer membrane proteins including porins and components of efflux pump systems. Porins typically control the diffusion of small metabolites including antibiotics, while efflux systems function via an energy-dependent mechanism to pump out unwanted toxic substances such as antibiotics through specific efflux pumps (Li et al., 2007, 2008; Vila et al., 2007; Jahandideh, 2013; Morita et al., 2014; Alcalde-Rico et al., 2016). Thus, the behaviors of these outer membrane proteins provide characteristic features of Gram-negative bacteria in response to antibiotics.

While many studies have investigated the action of individual outer membrane proteins during antibiotic stress, a proteomic approach reveals the dynamic features of the entire outer membrane proteome. Chloramphenicol-resistant *Escherichia coli* shared a similar outer membrane protein profile with the parental *E. coli* exposed to high concentration chloramphenicol (Morita et al., 2014). More importantly, several of the outer membrane proteins were up-regulated in response to one type of antibiotic but down-regulated to another. For example, OmpC was decreased in the differentially expressed outer membrane proteome of chloramphenicol-resistant *E. coli* but was unaffected in that of streptomycin-resistant *E. coli* (Li et al., 2007; Morita et al., 2014). differentially expressed outer membrane proteins overlapped in chloramphenicol-resistant and chlortetracycline-resistant *E. coli* cells, but OmpW was increased and decreased in chloramphenicol-resistant *E. coli* and in chlortetracycline-resistant *E. coli*, respectively (Lin et al., 2010; Morita et al., 2014). OmpC and AtpB were reversely expressed between chlortetracycline and nalidixic acid exposure in *E. coli* (Lin et al., 2012). Thus, the outer membrane proteome is largely associated with the resistance to the type of antibiotic, and may involve complex regulatory mechanisms (Li et al., 2008). This raises more questions in defining the role of each individual protein in an antibiotic-resistant outer membrane proteome.

Our investigation on the antibiotic-induced changes in the outer membrane proteome revealed that only a limited number of outer membrane proteins were changed in response to these antibiotics (Li et al., 2007, 2008; Lin et al., 2012), implying that a certain outer membrane protein response pattern may be present. Thus, we carried out a systematic study with three commonly used antibiotics to explore the possibility of an antibiotic-induced outer membrane protein pattern in *Edwardsiella tarda*. *E. tarda* is a Gram-negative bacilli that belongs to the Enterobacteriaceae family. This bacterium is known for causing diseases in humans, fish, and other species (Xie et al., 2015; Yang et al., 2015). In humans, *E. tarda* can cause gastroenteritis, colitis, dysentery-like disease, septicemia, and meningitis (Nelson et al., 2009; Kawai et al., 2011). As a fish pathogen, it is of particular importance to aquaculture and the fishing industry due to recent great economic loss caused by the bacterial pathogen (Xu and Zhang, 2014). Multidrug resistance *E. tarda* has been frequently isolated from fish and human (Wang et al., 2009; Kawai et al., 2011; Yu et al., 2012), presenting significant challenge for its control. Thus, the present study used the bacterium as a bacterial model to investigate outer membrane protein response pattern of antibiotic resistance. We identified six outer membrane proteins whose abundance were influenced by antibiotics, and were shared by antibiotic-resistant bacteria or multidrug resistance bacteria, thereby forming an antibiotic-induced pattern.

## Materials and methods

### Chemicals

Tryptic Soy Broth medium (TSB) and Lysogeny Broth medium (LB) were purchased from Huankai Biotech Limited (Guangzhou, China). All antibiotics including tetracycline, kanamycin, and chloramphenicol were purchased from Sangon Biotech Limited (Shanghai, China). DNA polymerase, T4 ligase, and restrictive endonucleases were obtained from Takara (Japan).

### Experimental design and statistical rationale

The objective of this experiment is to identify response patterns of outer membrane proteins to antibiotics by a comparative outer membrane sub-proteomics approach among bacterial strains with different antibiotic resistance and between clinically isolated strains with laboratory-generated antibiotic-resistant strains. Five clinically isolated *E. tarda* strains, LTB4, EIB202 (CCTCC M208068), WY28, WY37, and Et1, were used this study. Among these, LTB4 is susceptible to antibiotics and was sub-cultured in medium with kanamycin (KAN), tetracycline (TET), or/and chloramphenicol (CAP) to obtain LTB4-KAN, LTB4-TET, LTB4-CAP, LTB4-KAN/TET, LTB4-KAN/CAP, and LTB4-TET/CAP. EIB202, and Et1 were sub-cultured in KAN and CAP to obtain EIB202-KAN and Et1-CAP, respectively. For each sample, three biological repeats were cultured independently resulting in 39 samples. Outer membrane proteins were extracted from these samples and used for two-dimensional electrophoresis (2-DE)-based proteomics analysis. The abundance of individual protein spots was standardized based on their volume percentage of the total spots on the gel. Compared with control LTB4 (except for EIB202-KAN and Et1-CAP), EIB202 (EIB202-KAN), or Et1 (Et1-CAP), a spot with a greater than 2-fold change in abundance was considered a differentially expressed protein, which were identified by searching peptide mass fingerprint (PMF) mass spectrometry (MS) data against a protein database composed of purple bacteria and further validated by Western blot. Student's *t*-test was performed with Statistics Products and Service Solutions (SPSS) software version 11.5 (SPSS, Inc.) to determine the statistical significance of bacterial growth. Significant differences were considered present when *P* < 0.05.

### Bacterial strains and culture conditions

The five *E. tarda* strains above were kindly provided by professor YX Zhang of East China University of Science and Technology, China, professor Li Sun of the Institute of Oceanology of the Chinese Academy of Sciences, and professor XH Zhang of Ocean University of China. All strains were isolated from infected fish. Except for LTB4, others contain plasmids. In details, EIB202, WY28, and WY37 contain a 44 Kb plasmid encoding three drug-resistant genes, *tetA, tetR*, and *catA* (Wang et al., 2009; He et al., 2011) and Et1 contains an un-sequenced plasmid. All of the strains were cultured in TSB at 30°C and harvested when the optical density at 600 nm (OD_600_) is 1.0. The laboratory-generated antibiotic-resistant strains were obtained as described previously with a few modifications (Peng et al., 2005). In detail, the parental strains were propagated in a 1/2 MIC (minimal inhibitory concentration) of antibiotics (initial concentration: 1.56 μg/mL KAN, 0.31 μg/mL TET, or/and 0.5 μg/mL CAP) to select resistant strains with higher MIC, whose 1/2 concentration was used subsequently to culture those resistant strains. The strains were propagated likely to obtain strains with 10 MIC or higher MIC of the initial antibiotic concentration. Specifically, LTB4 was sub-cultured in KAN, TET, or/and CAP to obtain LTB4-KAN, LTB4-TET, LTB4-CAP, LTB4-KAN/TET, LTB4-KAN/CAP, and LTB4-TET/CAP; EIB202 and Et1 were sub-cultured in KAN and CAP to obtain EIB202-KAN and Et1-CAP, respectively.

### Determination of the minimum inhibitory concentration (MIC)

MIC was determined according to the Clinical & Laboratory Standards Institute (CLSI) procedure with a few modifications (CLSI, 2014). Briefly, overnight bacterial cultures were diluted 1:100 in fresh TSB medium and cultured at 30°C to an OD_600_ of 0.5. The log phase cells were diluted 1:1000 into each well of a 96-well microtiter polystyrene tray. The tray contained a series of 2-fold dilutions of antibiotics. After 24 h of incubation, the MIC was defined as the lowest concentration that inhibited visible growth. Three biological repeats with two technical replicates were carried out for each sample.

### Extraction of outer membrane proteins

Outer membrane proteins were isolated as previously described (Huang et al., 2006). Bacteria were grown overnight, diluted at 1:100 into fresh TSB medium without antibiotics and then harvested at OD_600_ = 1.0 by centrifugation at 4000 *g* for 15 min at 4°C. After being washed three times with sterile saline buffer (0.85% NaCl), the bacterial pellet was resuspended in 10 mL sonication buffer (50 mM Tris-HCl, pH 7.4). The solution was sonicated intermittently for periods of 7 s interspersed at 5 s intervals until the buffer clarified. Unbroken cells and cellular debris were removed by centrifugation at 5000 *g* for 20 min. The supernatant was further centrifuged at 100,000 *g* for 1 h at 4°C. The pellet was resuspended in 2% (W/V) sodium lauroyl sarcosinate (Sigma, USA) and incubated at room temperature for 30 min before additional ultracentrifugation. The concentration of outer membrane proteins was determined using a Bradford assay (Bradford, 1976) (Pierce, USA).

### 2-DE and MS

A linear 11-cm pH 3–10 immobilized pH gradient (IPG) (Bio-Rad, USA) was rehydrated overnight with 200 μL rehydration buffer (Bio-Rad, USA) (8 M urea, 2 M thiourea, 4% 3-[(3-Cholamidopropyl) dimethylammonio] propanesulfonate, 65 mM dithiothreitol (DTT) (Sigma, USA), 0.5% IPG buffer and 0.2% bromophenol blue) and loaded with 200 μg outer membrane proteins. The total isoelectric focusing time was 40,000 Vh and the maximum voltage was 8000 V (Multiphor II system) (Amersham, USA). After the first dimensional separation, the strips were sequentially equilibrated in DTT and iodine acetamide (4 M urea, 20% glycerol, and 10% SDS) (Sigma, USA) for 15 min, and then the IPG strips were transferred to 12% SDS-PAGE gels for the second dimension, which was followed by Coomassie Blue-R250 (Sigma, USA) staining. The 2-DE gels were scanned and processed using ImageMaster 5.0. Differentially expressed proteins were cut for trypsin digestion and mass spectrometric analysis as described before (Lin et al., 2008). All of the MALDI analyses were performed with a fuzzy logic feedback control system (Reflex III MALDI-TOF system, Bruker, Karlsruhe, Germany) equipped with delayed ion extraction. MS peaks were selected between 800 and 3000 D and were filtered with a signal-to-noise ratio greater than 15 to exclude masses derived from trypsin autolysis. The MS data were interpreted and processed using Flexanalysis 3.0 (Bruker Daltonics), and the spectra obtained for each spot were combined and entered into the MASCOT search engine (V2.3, Matrix Science, London, U.K.) using Biotools 3.1 (Bruker Daltonics). The following parameters were used in the search: NCBI in SwissProt (http://www.matrixscience.com), one missed cleavage site, carbamidomethyl as a fixed modification of cysteine and oxidation of methionine as a variable modification, MS tolerance of 100 ppm. Known contaminant ions (keratin) were excluded. A 95% confidence level threshold was used for the MASCOT protein scores (NCBInr 20160717; 90832692 sequences, 33437327307 residues). Proteins with a MASCOT score higher than 78 were identified for peptide mass fingerprinting. Other identification criteria included at least 42% sequence coverage for MS. The resulting data are listed in Supplementary Tables 1, 2.

### Western blotting

Proteins were separated by SDS-PAGE and then transferred to nitrocellulose membranes (GE healthcare, USA). The membrane was blocked for 60 min with 5% skim milk in Tris-buffered saline plus 0.05% Tween-20 (TBST). After incubation with the mouse polyclonal antibodies anti-EvpB, anti-TolC, anti-ETAE_1826, anti-ETAE_2675, anti-ETAE_1826, anti-OmpS2, or anti-OmpA as the primary antibodies (1:500–2000 dilution) and anti-mouse-IgG-HRP (1:2000 dilution) (Boshen, Xiamen) as the secondary antibody, the positive bands were visualized with 3,3′-diaminobenzidine.

### Co-immunoprecipitation (Co-IP)

Co-IP was carried out as described previously (Pan et al., 2011). Mouse antisera against ETAE_p037 or ETAE_p044 were purified with 33% saturated ammonium sulfate and incubated with a mixture of recombinant ETAE_p037 or ETAE_p044 with *E. tarda* outer membrane proteins for 12 h at 4°C on a gentle shaker. Then, nProtein A Sepharose 4 Fast Flow (Amersham Biosciences Corp., USA) was added and the solution was incubated for 12 h at 4°C on a gentle shaker. The nProtein A Sepharose 4 Fast Flow was collected by centrifugation at 2000 *g* for 3 min, cleaned with pH 7.0 Tris-HCl buffer six times for 10 min, incubated in pH 2.4 glycine-HCl buffer for 2 h, adjusted to pH 7.4 with 0.1 M Tris and then subjected to SDS-PAGE. The distinct bands on the gels were analyzed using MALDI/TOF-TOF analysis. OmpF2 and OmpA were further identified by Western blot using mouse anti-OmpF2 and anti-OmpA as the primary antibodies and anti-mouse-IgG-HRP as the secondary antibody as described above.

### Pattern recognition analysis

Pattern recognition analysis was carried out as described previously (Zhang et al., 2012; Peng et al., 2015). The protein data are presented as the mean ± *SD*. Hierarchical clustering was performed on the log-transformed normalized data using the R platform with the gplots package (https://cran.r-project.org/web/packages/gplots/index.html) and the distance matrix. ICA was used to discriminate sample patterns, identify the proteins associated with the antibiotics and minimize the influence of interindividual variation.

### Expression of OmpS2 and OmpA in LTB4 exposed to TET

LTB4 cells grown to an OD_600_ at 1.0 were collected and exposed to 0.2 μg/mL TET for 1 h. The resulting bacteria were collected and used for Western blotting to detect the abundance of OmpS2 and OmpA using mouse anti-OmpF2 and anti-OmpA as the primary antibodies, respectively, and anti-mouse-IgG-HRP as the secondary antibody as described above.

### Antimicrobial susceptibility with survival assay

A survival capability assay was performed as described previously (Li et al., 2008). In brief, inoculums of BL21-32a-OmpS2, which was generated by cloning the full length of OmpS2 using primers (forward 5′-GC*GAATTC*ATGTGTTTACTTCGACAG-3′, *EcoRI* and reverse 5′-GC*CTCGAGT*TAGAACTGGTAGACCAGA-3′ XhoI) from EIB202 genomic DNA, cloned into pET32a expression vector and transformed into BL21, were cultured separately overnight and diluted 1:1000 into 5 mL fresh LB medium with 0.02 mM IPTG. Two cultures contained TET, and the other two were used as controls without the drug. Bacteria were cultured at 28°C on a gentle shaker (150 rpm), and the OD_600_ of the bacterial cultures was detected at 8 h. Survival capability was calculated by dividing the OD of the cultures with TET by the OD of the cultures without the antibiotic and termed the survival rate of bacterial strains cultured in medium with the drug. The experiment was repeated at least three times. The differences in survival rates were analyzed with SPSS. Differences were considered significant if *p* < 0.05.

## Results

### Evaluation of the antibiotic resistance of five clinically isolated *E. tarda* strains

**Figure 1:**
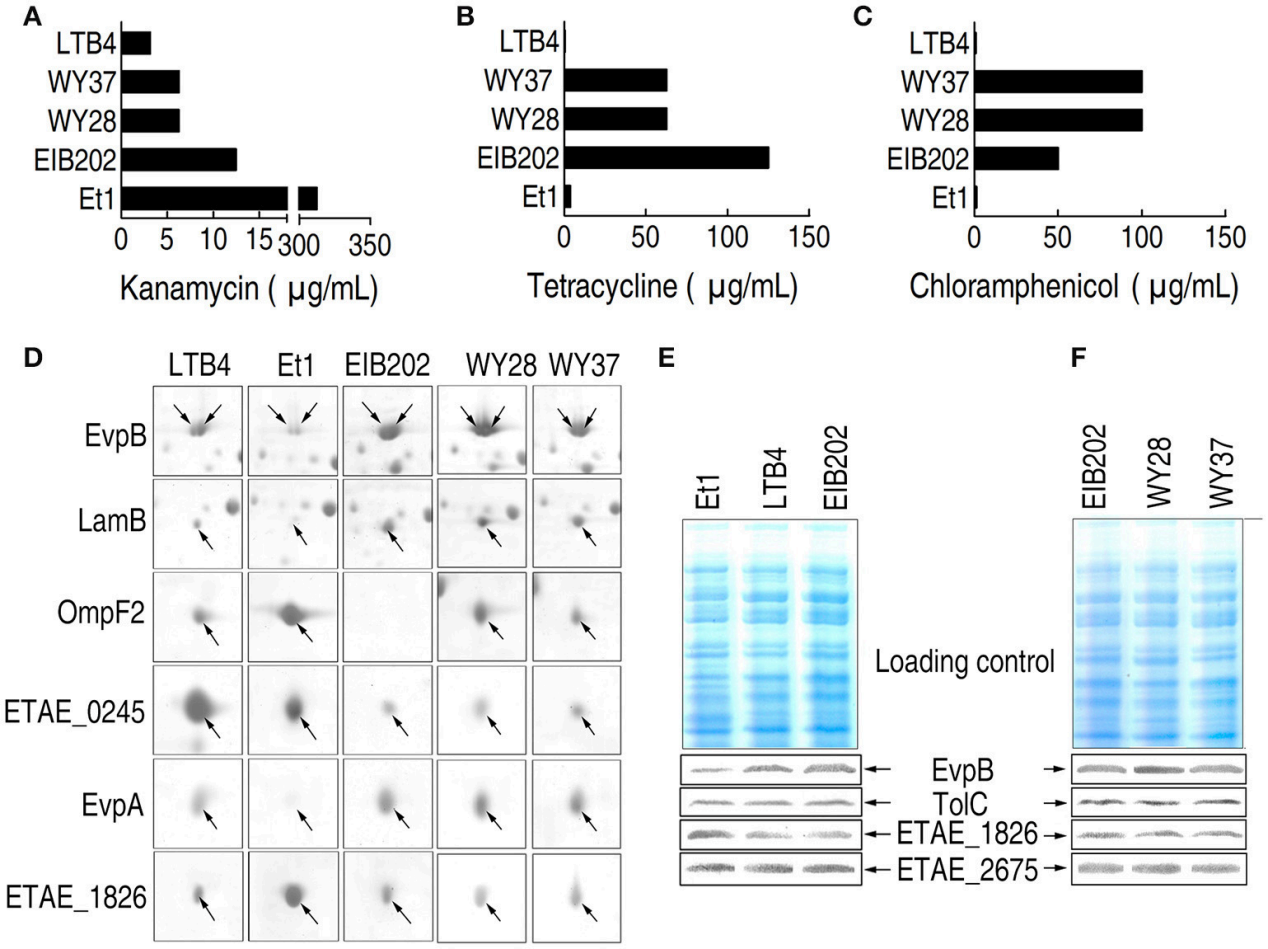
**MIC and 2-DE-based proteomics for the analysis of outer membrane proteins with differential abundance in five clinically isolated ***E. tarda*** strains. (A–C)** MICs for kanamycin **(A)**, tetracycline **(B)**, and chloramphenicol **(C)** in the five clinically isolated *E. tarda* strains. **(D)** Enlarged image showing six outer membrane proteins with differential abundance in the five clinically isolated strains. **(E,F)** Western blotting to validate the differential abundance of EvpB and ETAE_1628, using unchanged TolC and ETAE_2675 as controls.

The MICs of the five clinically isolated strains EIB202, LTB4, WY28, WY37, and Et1 to KAN, TET, and CAP are determined (Figures 1A–C). Et1 exhibited high KAN resistance but low TET and CAP resistance while EIB202, WY28, and WY37 exhibited high TET and CAP resistance but low KAN resistance. LTB4 was susceptible to all three antibiotics and thus served as the negative control.

### Establishment of outer membrane protein profiles associated with antibiotic resistance

2-DE proteomics was used to characterize the differential abundance of outer membrane proteins in the five clinically isolated *E. tarda* strains (Supplementary Figures 1A, 2). The differentially expressed proteins from the five strains are summarized in Figure 1D; Supplementary Figures 2A, 3; Supplementary Table 1. The expression change was further validated by Western blot (Figures 1E,F). Thus, the six proteins represent the arsenal for outer membrane protein being associated with antibiotic resistance.

### Identification of KAN-, TET-, and CAP-induced outer membrane protein patterns

**Figure 2:**
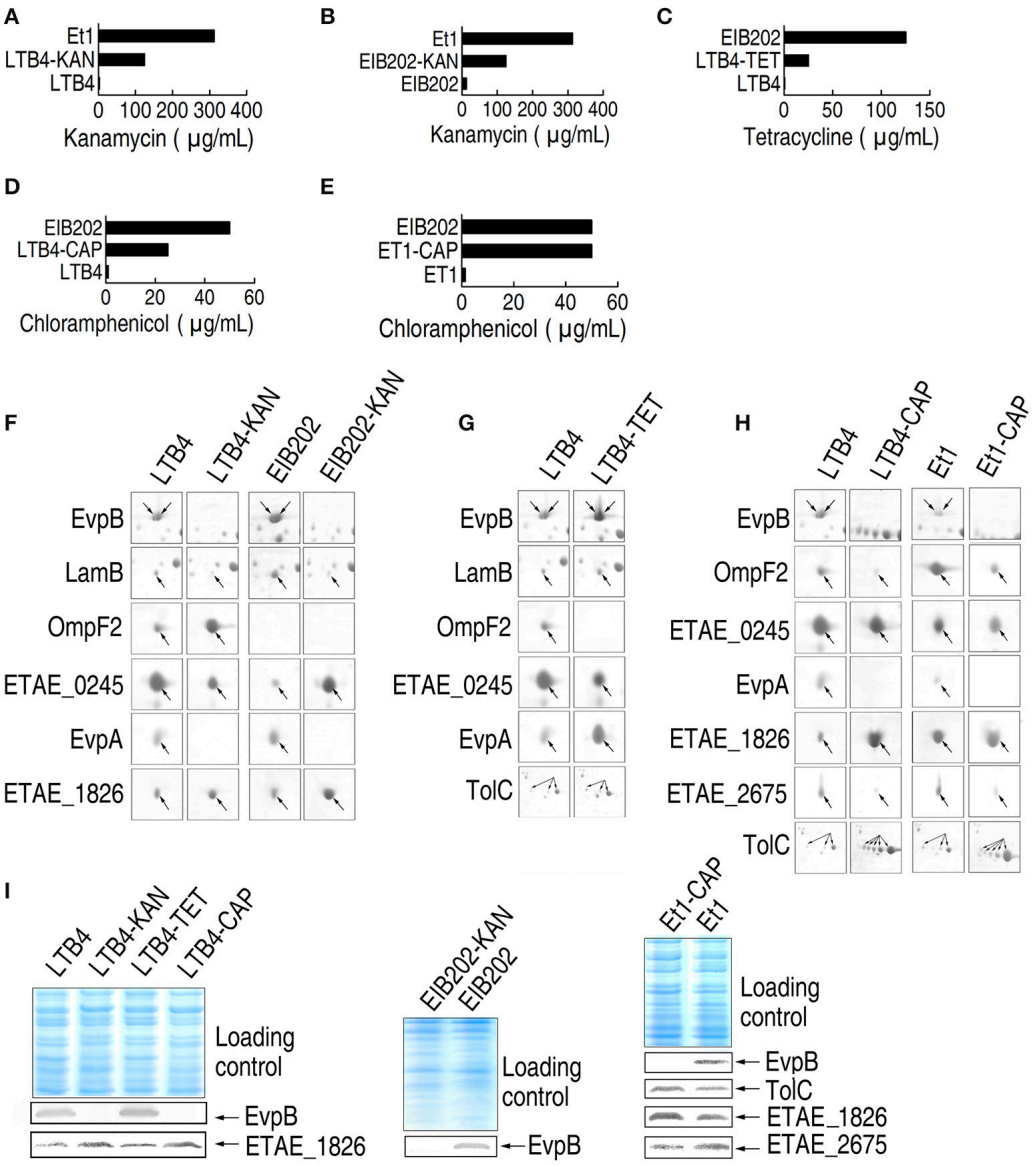
**MIC and 2-DE-based proteomics for the analysis of outer membrane proteins with differential abundance in five laboratory-generated antibiotic-resistant ***E. tarda*** strains. (A–E)** MICs for the laboratory-generated antibiotic-resistant *E. tarda* strains. **(F–H)** The enlarged maps for the spots with differential abundance in KAN (kanamycin)-resistant strains **(F)**, TET (tetracycline)-resistant strains **(G)**, and CAP (chloramphenicol)-resistant strains **(H)**. **(I)** Western blotting to validate the differential abundance of proteins.

To establish the relationship between the outer membrane proteome and antibiotic resistance, we generated specific antibiotic-resistant strains. LTB4 was propagated with KAN, TET, or CAP to generate KAN-, TET-, and CAP-resistant strains, which were named LTB4-KAN, LTB4-TET, and LTB4-CAP, respectively. Meanwhile, EIB202 was propagated with KAN to obtain EIB202-KAN and Et1 was propagated with CAP to generate Et1-CAP. The MICs of the laboratory-generated antibiotic-resistant strains were lower (2.5-folds for LTB4-KAN and EIB202-KAN, 5-folds for LTB4-TET, 2-folds for LTB4-CAP), or equal (for ET1-CAP) to those of the clinically isolated antibiotic-resistant strains (Figures 2A–E).

We used 2-DE-based proteomics to characterize the differential abundance of outer membrane proteins in the five laboratory-generated strains. The results showed that six, five, six, seven, and seven proteins exhibited differential abundance in LTB4-KAN, EIB202-KAN, LTB4-TET, LTB4-CAP, and Et1-CAP compared to their parental strains, respectively. The abundance of EvpB, LamB, and EvpA was lower in LTB4-KAN and EIB202-KAN compared to their parental strains and the abundance of OmpF2 and ETAE_1826 was higher in LTB4-KAN and EIB202-KAN compared to their parental strains (Figure 2F; Supplementary Figures 1B, 2B, 3; Supplementary Table 1), which was consistent with the differential abundance of outer membrane proteins in Et1 compared with LTB4. Second, the abundance of OmpF2 and ETAE_0245 was lower in LTB4-TET than its parental strain, and the abundance of EvpB, LamB, EvpA, and TolC was higher in LTB4-TET than its parental strain (Figure 2G; Supplementary Figures 1B, 2C, 3). The same difference was also observed between LTB4 and EIB202. Finally, EvpB, OmpF2, ETAE_0245, EvpA, and ETAE_2675 were lower and ETAE_1826 and TolC were increased (Figure 2H; Supplementary Figures 1B, 2D, 3); similar differences were observed between LTB4 and LTB4-CAP and between Et1 and Et1-CAP. In addition, these changes in abundance were validated by Western blot (Figure 2I).

### Establishment of KAN-, TET- and CAP-induced outer membrane protein patterns

**Figure 3:**
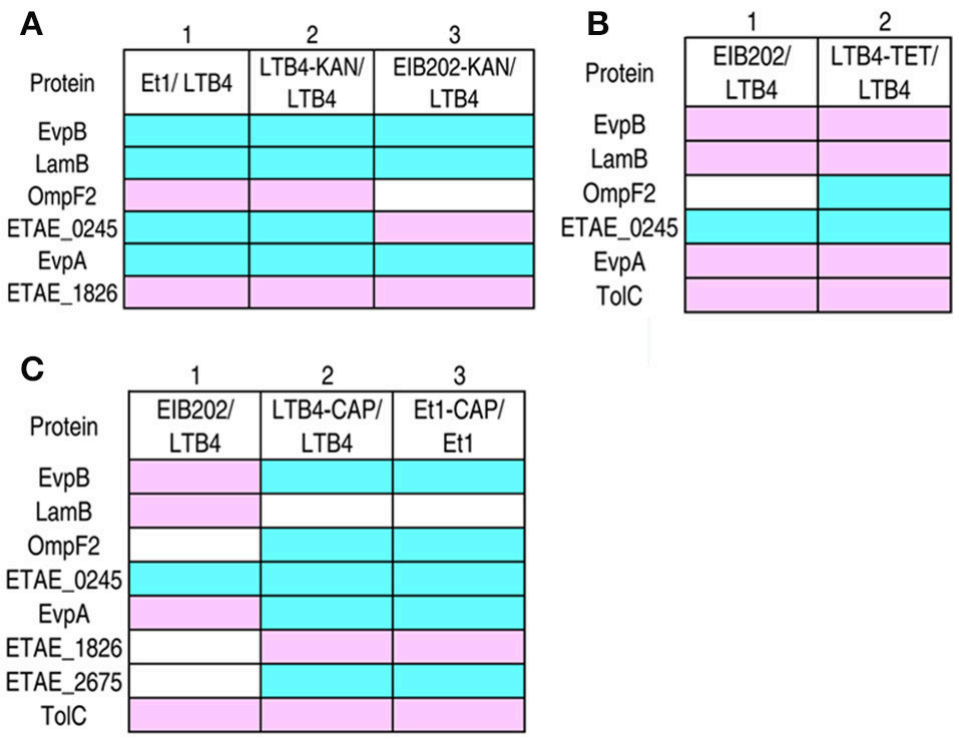
**Comparison of the outer membrane proteins with differential abundance among different antibiotic-resistant ***E. tarda*** strains**. Blue, decrease; pink, increase; white, no change. **(A)** KAN-resistant strains. **(B)** TET-resistant strains. **(C)** CAP-resistant strains.

To characterize the KAN-resistant outer membrane protein pattern, all of the KAN-resistant strains were compared, and the results were summarized in Figure 3A. In group I, Et1 was compared with LTB4, and 6 proteins were identified. In this group, the abundance of EvpB, LamB, ETAE_0245, and EvpA was decreased and the abundance of OmpF2 and ETAE_1826 was increased. In groups II and III, LTB4-KAN and EIB202-KAN were compared with LTB4 and EIB202, respectively. The change in the abundance of the proteins was consistent with the changes in group I, with the exception of ETAE_0245 in group III (OmpF2 is not present in EIB_202). Et1 is resistant to KAN, and LTB4 is susceptible to KAN. The differentially expressed proteins in these two strains could be the key proteins involved in KAN resistance. The last two groups, LTB4 and EIB202, were susceptible to KAN and acquired KAN resistance, respectively. The changes in abundance of outer membrane protein in the last two groups were consistent with the changes in group I (Figure 3A). Thus, the variation of these proteins was the KAN-resistant outer membrane protein pattern.

Similarly, the TET- and CAP-resistant outer membrane protein patterns were summarized in the same way and are shown in Figures 3B,C, respectively. Figure 3B shows the comparison of EIB202 and LTB4, which identified five differentially expressed proteins: EvpB, LamB, EvpA, and TolC were higher in EIB202 and ETAE_0245 was lower in EIB202. EIB202 is resistant to TET, so these proteins could be important in TET resistance. Although the abundance of these proteins was also altered in KAN-resistant strains, the changes in the abundance of these proteins were different for different antibiotics (with the exception of ETAE_0245). In the second group shown in Figure 3B, the comparison between the TET-resistant strain and the parental strain identified six proteins with altered abundance. Five of these proteins were also present in group I, and the last protein was OmpF2, which is absent from EIB202. The variation in these six proteins was the TET-resistant pattern.

In Figure 3C, group I indicates those proteins exhibiting altered abundance in the native CAP-resistant strain and a CAP susceptible strain. There were five proteins with altered abundance: EvpB, LamB, and EvpA were higher in the CAP-resistant strain, but OmpF2 and ETAE_0245 were lower in the same strain. Groups II and III compared LTB4-CAP and Et1-CAP with their parental strains. Although the proteins exhibiting altered abundance in the laboratory-generated CAP-resistant strains and native CAP-resistant strains were not the same, the differential expressed proteins of the two laboratory-generated CAP-resistant strains were the same. Of the eight differentially expressed proteins, ETAE_0245 and TolC were consistent across all three groups. In addition, the profile of OmpF2 was also similar due to the absence of this protein from EIB202. These results indicated that these proteins are important for CAP resistance.

EIB202 is resistant to both TET and CAP and exhibits a TET MIC 200 times greater than the TET MIC of LTB4; however, the EIB202 CAP MIC is only 50 times greater than the CAP MIC of LTB4. This finding suggests that resistance to one antibiotic may be influenced by resistance to another antibiotic, leading to an altered protein pattern that is inconsistent between the clinically isolated antibiotic-resistant strain and the laboratory-generated CAP-resistant strains. However, seven proteins were identified from the differentially expressed proteins of the two laboratory-generated CAP-resistant strains in groups II and III: ETAE_0245, TolC, OmpF2, EvpA, EvpB, ETAE_1826, and ETAE_2675 were important to CAP resistance, and the variation in these seven proteins was the CAP resistance pattern.

### Antibiotic-induced responsive patterns of outer membrane proteins in double drug-resistant strains

**Figure 4:**
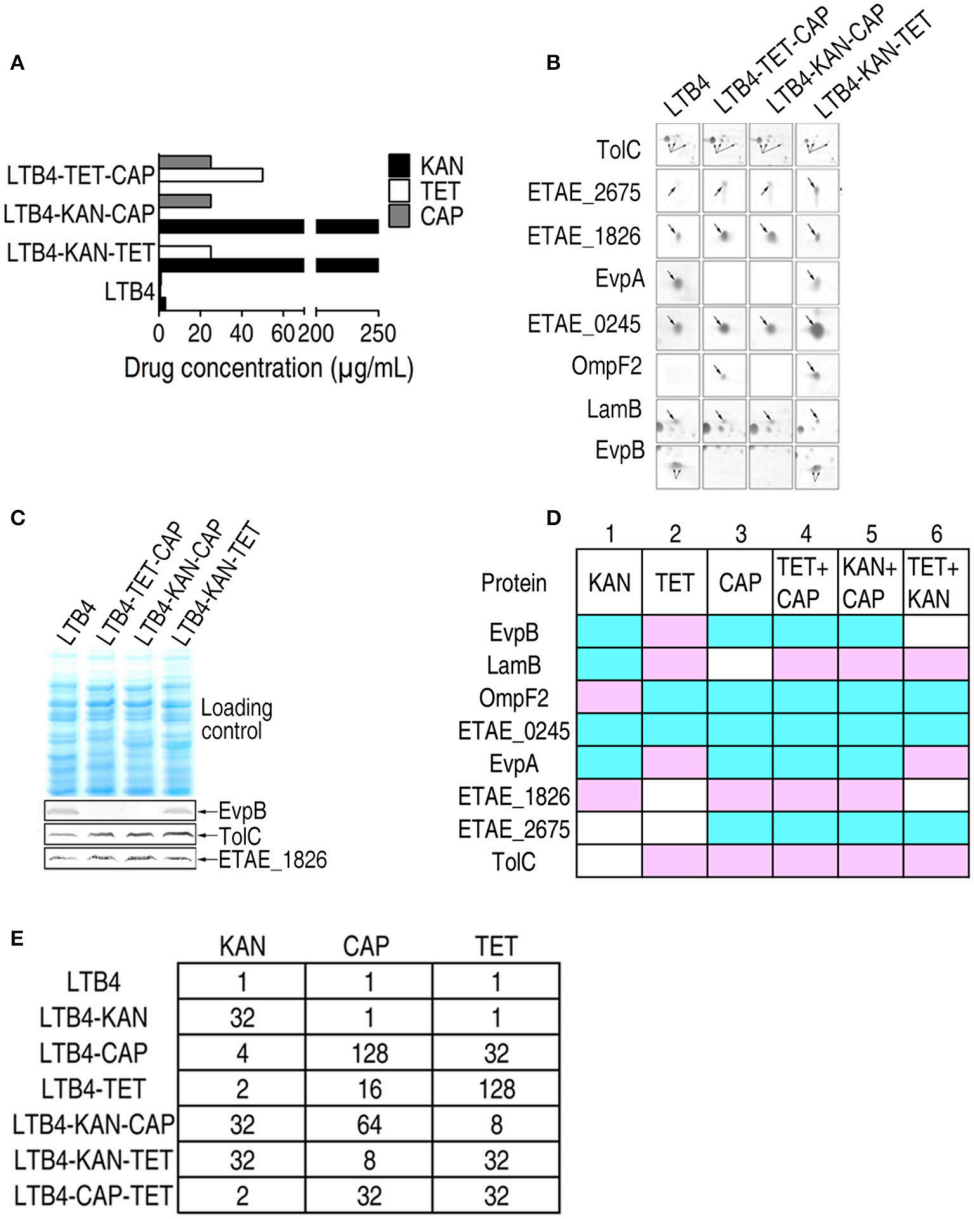
**MIC and 2-DE-based proteomics for the identification of outer membrane proteins with differential abundance among three laboratory-generated double antibiotic-resistant strains**. Arrows indicates the spots of interest. **(A)** MICs for the laboratory-generated double antibiotic-resistant *E. tarda* strains. **(B)** Enlarged maps showing the differentially expressed outer membrane proteins. **(C)** Western blot for the detection of EvpB, TolC, and ETAE_1826 in the three double antibiotic-resistant strains. **(D)** Comparison of the laboratory-generated antibiotic-resistant *E. tarda* strains. Blue, decrease; pink, increase; white, no change. **(E)** KAN (kanamycin), CAP (chloramphenicol), and TET (tetracycline) MICs for the six laboratory-generated strains compared with LTB4.

The three antibiotics KAN, TET, and CAP had different effects on the outer membrane proteins and even exhibited reversed effects on some proteins. To investigate the combinatorial effects of two antibiotics on outer membrane proteins, LTB4 was cultured in two of the three antibiotics. The resulting strains were LTB4-KAN-TET, LTB4-KAN-CAP, and LTB4-TET-CAP (Figure 4A). In the double drug-resistant strains, the MIC for each antibiotic was similar to the MIC for the clinically isolated antibiotic-resistant strain.

By 2-DE, eight proteins exhibited altered abundance in all three of the double drug-resistant strains. In the LTB4-TET/CAP and LTB4-KAN/CAP strains, EvpB, OmpF2, ETAE_0245, EvpA, and ETAE_2675 decreased and LamB, ETAE_1826, and TolC increased compared to the LTB4 strain. This change is quite similar to the CAP-induced response pattern, suggesting that CAP resistance pattern is dominant over TET and KAN resistance patterns. In the LTB4-KAN/TET strain, LamB, EvpA, and TolC increased while OmpF2, ETAE_0245, and ETAE_2675 decreased compared to the LTB4 strain; this pattern is similar to the TET-reduced response pattern (Figure 4B; Supplementary Figure 2E). Therefore, this finding suggests that TET resistance pattern is dominant over KAN resistance pattern. The differences in EvpB, TolC, and ETAE_1826 were further validated by Western blot (Figure 4C).

### The comparison of the differentially expressed proteins among antibiotic-resistant strains

As summarized in Figure 4D, the comparison of the six laboratory-generated antibiotic-resistant strains identified eight differentially expressed proteins, including EvpB, LamB, OmpF2, ETAE_0245, EvpA, ETAE_1826, ETAE_2675, and TolC. All of the proteins except for ETAE_0245 showed variation in all six of the strains. Interestingly, the presence of double antibiotic resistance significantly changed the outer membrane protein pattern. We hypothesized that resistance to one type of antibiotic could be influenced by another type of antibiotic. In rows 3–5, seven of the eight differentially expressed proteins were the same. These changes correspond to CAP-induced resistance because both the single and double drug-resistant strains (LTB4-TET-CAP and LTB4-KAN-CAP) showed similar changes. Thus, CAP-induced resistance pattern is dominant over the other types of resistance patterns and affects the outer membrane proteome in these drug-resistant strains. Accordingly, five of the differentially expressed proteins were the same in rows 2 and 6, so TET-induced resistance pattern was dominant over KAN-induced resistance pattern in affecting the outer membrane proteome. However, the proteins in row 1 were different from those in any other rows, suggesting the KAN resistance pattern without CAP and TET resistance. In summary, if outer membrane protein patterns were differentially influenced by two antibiotics, the influence imposed by one of the antibiotics may be dominant, with the hierarchical relationship being CAP > TET > KAN.

Furthermore, LTB4-KAN was susceptible to CAP and TET with the same MIC as LTB4, whereas LTB4-CAP and LTB4–TET were resistant to KAN with a 4-fold higher MIC than LTB4. Similarly, LTB4-CAP and LTB4-TET exhibited the same MIC to the corresponding drug, but LTB4-CAP displayed an MIC of 32 to TET while LTB4-TET exhibited an MIC of 16 to CAP. Interestingly, in the double antibiotic resistance, the MIC of LTB4-CAP-TET to KAN was 2, which was lower than that of LTB4-KAN-CAP to TET and LTB4-KAN-TET to CAP, indicating an effect of double antibiotic resistance on antibiotic sensitivity (Figure 4E).

### Cluster analysis of clinically isolated and laboratory-generated antibiotic-resistant strains

**Figure 5:**
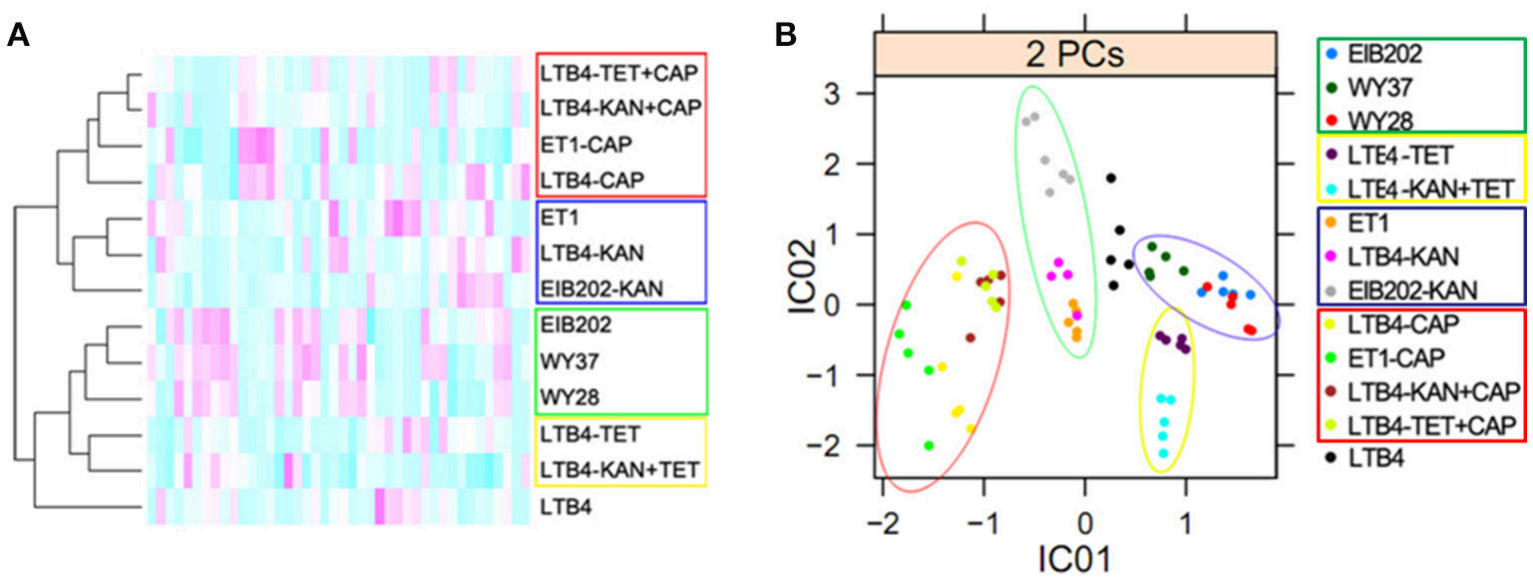
**Bioinformatics analysis of outer membrane proteins in 15 clinically isolated and laboratory-generated strains**. The red circles indicate the CAP-resistant strains; the green circles indicate the KAN-resistant strains; the yellow circles indicate the TET-resistant strains; and the blue circles indicate EIB202, WY37, and WY28, which have the similar resistance to antibiotics. **(A)**, Clustering analysis. Red to green colors corresponds to low to high abundance. **(B)**, Independent component analysis. Significant differences between the data points are shown. One data point represents one subject.

All the spots in the 2-DE maps of the 13 strains were analyzed with cluster analysis, which showed that: (1) LTB4, the control strain, was individually separated; (2) Four CAP-resistant strains, ET1-CAP, LTB4-CAP, and two double resistant strains, were clustered together; (3) LTB4-TET and LTB4-KAN-TET were clustered together; (4) ET1, LTB4-KAN, and EIB202-KAN were clustered together; (5) The other three strains, EIB202, WY28, and WY37, which had similar drug-resistant features and outer membrane proteins, were clustered together (Figure 5A). The relative locations of the clustered strains were in accordance with our proposed hierarchy of antibiotic resistance, which was CAP > TET > KAN.

The ICA analysis showed a result similar to the cluster analysis. The CAP-resistant strains were divided into a category in the red circle; the KAN-resistant strains were in the green circle; the TET-resistant strains were in the yellow circle; and EIB202, WY37, and WY28 were in the blue circle. Factor IC01 separated CAP and KAN resistance from the other three types of resistance, and factor IC02 separated CAP and TET from the others (Figure 5B). The leading antibiotic determined the categories of the ICA analysis.

### Plasmid-encoded antibiotic-resistance alters the outer membrane protein pattern

**Figure 6:**
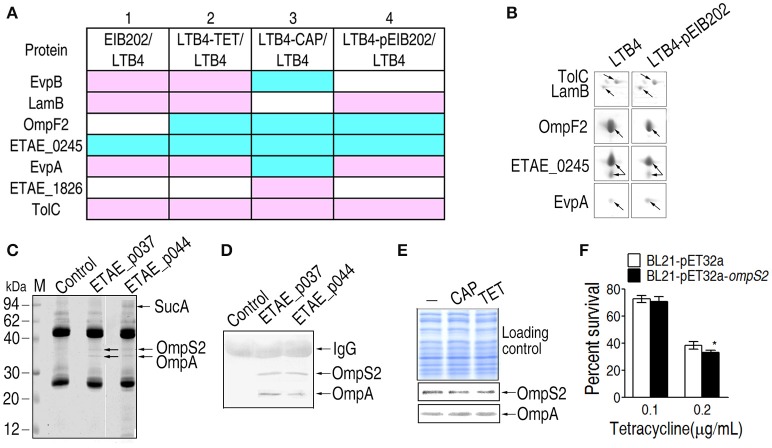
**Differentially expressed outer membrane proteins and their roles in LTB4-pEIB202. (A)** Enlarged maps for the differentially-expressed proteins in LTB4-pEIB202. Blue, decrease; pink, increase; white, no change. **(B)** Comparison of the differentially-expressed outer membrane proteins in LTB4-pEIB202 and other antibiotic-resistant *E. tarda* strains. **(C,D)** Co-IP for the detection of the proteins interacting separately with ETAE_p037 and ETAE_p044. Anti- ETAE_p037 and anti-ETAE_p044 were separately incubated with mixtures of recombinant ETAE_p037 or ETAE_p044 and *E. tarda* outer membrane proteins. The interacting proteins were identified by MALDI/TOF (OmpS2, OmpA, and SucA, which are indicated with arrows) and Western blot **(D). (E)** Western blot detecting the differential abundance of OmpS2 and OmpA in LTB4 cells exposed to CAP (chloramphenicol) and TET (tetracycline). **(F)** Percent survival of OmpS2-overexpressing LTB4 cells exposed to TET. *p < 0.05 using Student's *t*-test.

The EIB202 plasmid encodes three drug-resistant genes, *tetA, tetR*, and *catA* (Kawai et al., 2011), which are responsible for bacterial resistance to TET and CAP. With this plasmid, the MIC for TET was 200 times more than that for LTB4, but the MIC for CAP was only 50 times higher than that for LTB4. Because the outer membrane proteins of the TET-resistant strains had abundance profiles more similar to EIB202 than LTB4 (Figure 6A), a potential link between plasmid-acquired antibiotic resistance and outer membrane protein-related resistance may exist. Therefore, the plasmid isolated from EIB202 was introduced into LTB4, generating strain LTB4-pEIB202. Five differentially expressed proteins, including TolC, LamB, EvpA, OmpF2, and ETAE_0245, were identified by comparing 2-DE maps for LTB4-pEIB202 and LTB4. TolC, LamB, and EvpA were higher in LTB4-pEIB202 compared to LTB4, but OmpF2 and ETAE_0245 were lower (Figures 6A,B). This result demonstrates that antibiotic resistance conferred by plasmid can also influence the abundance of certain outer membrane proteins, forming a plasmid-acquired resistance pattern, which is similar to the corresponding antibiotic-induced pattern.

### The regulation of outer membrane protein abundance in response to plasmid and antibiotics

The differentially expressed proteins of the plasmid-acquired antibiotic-resistant strain and the laboratory-generated antibiotic-resistant strains are summarized in Figure 6A. Row 1 shows the comparison between EIB202 and LTB4 and rows 2 and 3 show the comparisons between the laboratory-generated TET and CAP antibiotic-resistant strains and their parental strains, respectively. Row 4 shows the comparison between the plasmid-acquired antibiotic-resistant strain and its parental strain. The differentially expressed proteins in Figures 4B,C also appear here for antibiotic inducement (row 2 or 3) and plasmid influence (row 4). The changes in TolC, OmpF2, and ETAE_0245 were the same in all four rows. Moreover, LamB and EvpA were the same with TET-induced resistance. These results show that the plasmid not only regulates the abundance of outer membrane proteins but also contributes to antibiotic resistance, generating synergism in antibiotic resistance.

Furthermore, Co-IP was used to probe the interaction between the proteins encoded by the plasmid and the outer membrane proteins. Two proteins, OmpS2 and OmpA, interact with ETAE_p037 (tetracycline repressor protein R class A variant); and three proteins, SucA, OmpS2, and OmpA, interact with ETAE_p044 (chloramphenicol O-acetyltransferase). OmpS2 is a porin that allows passive diffusion across the membrane and OmpA is an outer membrane antigenic protein (Moreno-Eutimio et al., 2013; Wang et al., 2016). SucA is a subunit of the alpha-ketoglutaric dehydrogenase complex, which catalyzes alpha-oxoglutarate to succinic acid (Liu et al., 2015). Furthermore, the presence of OmpS2 and OmpA was validated by Western blot (Figure 6C). We further showed the lower OmpS2 and the higher LTB4 in response to TET (Figures 6D,E). *E. coli* overexpression OmpS2 show decreased survival in the survival capability assay (Figure 6F).

## Discussion

KAN, TET, and CAP exert antimicrobial activities through a common mode of action, i.e., inhibition of protein synthesis, but they belong to different classes of antibiotics, as aminoglycoside, tetracyclines, and chloramphenicol, respectively. Correspondingly, bacterial antibiotic-resistant mechanisms to the three classes of antibiotics are class-specific (Labby and Garneau-Tsodikova, 2013; Grossman, 2016). The most prevalent mechanism of resistance to the aminoglycoside family of antibiotics is the action of aminoglycoside-modifying enzymes (Labby and Garneau-Tsodikova, 2013). Resistance to tetracycline is attributed to ribosomal protection that is mediated by chromosomal or plasmidborne *tetM* or *tetO* genes, and plasmids that is mediated by *tetK* and *tetL* genes (Grossman, 2016). Key mechanisms for CAP resistance are efflux pumps such as *floR* and *cmlA*, as well as inactivating enzymes such as chloramphenicol acetyl-transferase *cat1* (Grossman, 2016). Besides these, evidence has indicated that bacterial outer membrane proteins play key roles in antibiotic resistance (Xu et al., 2006; Zgurskaya et al., 2015; Urfer et al., 2016). However, it is unknown whether the outer membrane proteins behave in a specific way in response to antibiotics. The present study specifically investigated the outer membrane proteome of *E. tarda* strains with different types of antibiotic resistances including KAN, TET, and CAP resistance patterns. These patterns were further validated in double drug-resistant strains and plasmid-conferred antibiotic-resistant strain. These results indicate an antibiotic-resistance response pattern of outer membrane proteins; this pattern is related to antibiotic resistance. Further studies are required to understand the mechanism about the relationship and the impact of possible multiple mutations generated in laboratory step-wise selection of these strains.

Eight outer membrane proteins were identified as being associated with different types of antibiotic resistances including, EvpB, LamB, OmpF2, ETAE_0245, EvpA, ETAE_1826, ETAE_2675, and TolC. Interestingly, the roles of the eight proteins were not reported to be associated with antibiotic resistance in *E. tarda*. But the roles of five of the proteins or their homologs have been reported. EvpA and EvpB are vital for *E. tarda* pathogenesis as components of a type III secretion system (Rao et al., 2004; Chakraborty et al., 2011). The functions of OmpF2, LamB, and TolC were poorly understood in *E. tarda*, which were only revealed in other bacteria. The transcription of OmpF2 was increased when medium osmolality changed in *ompR* deficient mutations, implying the role of OmpF2 in maintaining the membrane permeability during stress (Gao et al., 2011). LamB is an important bacterial porin that forms complex with Odp1, and confers antibiotic resistance in *E.coli* (Lin et al., 2014). Depletion of LamB in *Klebsiella peneumoniae* make it susceptible to antibiotics (Garcia-Sureda et al., 2011). Inactivation of TolC makes enteric bacteria sensitive to a broad range of antimicrobial agents including antibiotics, detergents, dyes, organic solvent and others. More importantly, the functions of TolC are tightly associated with efflux pumps like AcrAB in clinical *E. coli* isolates. Thus, the study from homologies of TolC implied TolC is a critical protein in response to antibiotics, and not astonish to identify this protein in antibiotic-resistant bacteria (Sulavik et al., 2001; Zgurskaya et al., 2011; Li et al., 2016). Of notice, whether the six proteins were regulated by master regulators like CpxR or they were involved in distinct pathways await further investigations (Li et al., 2008; Xiong et al., 2010; Huang et al., 2016). Blast analysis shows that ETAE_2675, ETAE_1862 and ETAE_0245 share 54, 75, and 59% identity with a virulent protein (WP_047782217.1) of *Pragia fontium*, outer membrane protein (EHM38899.1) of *Hafnia alvei* ATCC 51873 and membrane protein (WP_046459876.1) of *Hafnia alve*, but further function information is not available. Their roles also need further research on drug-resistant *E. tarda*.

Although these outer membrane proteins are associated with drug resistance, the change of abundance of these proteins was not the same for each antibiotic and was even opposite in some cases. Comparison of the 2-DE maps for single drug-resistant and double drug-resistant strains revealed that outer membrane proteins were differentially regulated by different classes of antibiotics. If two antibiotics affect the same protein, the change of abundance of the outer membrane proteins would be dominated by one of the antibiotics. Based on our tested antibiotics, the hierarchical relationship for the three antibiotics is CAP > TET > KAN. The cluster analysis and ICA analysis of the 2-DE maps showed LTB4 in an independent category as the susceptible strain and the CAP-, TET-, or KAN-resistant strains separately divided into different categories depending on the “dominant pattern.” These results are helpful in understanding multidrug resistance.

Antibiotics can regulate the abundance of outer membrane proteins, but the outer membrane proteins of EIB202, which has a drug resistance plasmid (Wang et al., 2009), still show the drug resistance characteristics of the LTB4 strain in medium without an antibiotic. To solve this question, the EIB202 plasmid was transferred into the LTB4 strain, generating a plasmid-acquired antibiotic-resistant strain. The outer membrane proteins in the 2-DE maps of the plasmid-acquired antibiotic-resistant strain showed some of the characteristics of the laboratory-generated antibiotic-resistant strains when the plasmid-acquired antibiotic-resistant strain was cultured in medium without an antibiotic, suggesting that the plasmid could regulate the abundance of outer membrane proteins, and indicating synergism between plasmid-acquired and intrinsic drug resistance. The functional assay showed that OmpS2, which interacts with plasmid-encoding proteins, was lower under TET stress, and the expression of OmpS2 increased TET sensitivity. Thus, plasmids encode proteins that can directly influence the drug resistance of bacteria and regulate the expression of outer membrane proteins without antibiotic inducement.

In summary, the present study reveals an antibiotic-induced responsive pattern of outer membrane proteins in antibiotic resistance bacteria cultured in medium without an antibiotic. The finding highlights the way to design outer membrane protein arrays to identify diversity patterns of multidrug resistance as a biomarker of clinical medication.

## Author contributions

XP, BP, and HL conceptualized the idea and designed the experiment; CW, BP, YS, JY, MY, and MJ performed the experiment; XP, BP, CW, and HL interpreted the data. XP and BP wrote the manuscript.

### Conflict of interest statement

The authors declare that the research was conducted in the absence of any commercial or financial relationships that could be construed as a potential conflict of interest.
