# Supplementary material for: Outer Membrane Proteins form Specific Patterns in Antibiotic-Resistant Edwardsiella tarda
Source: Front Microbiol. 2017 Feb 2;8:69. doi: 10.3389/fmicb.2017.00069 (PMC5288343; doi:10.3389/fmicb.2017.00069)
Supplement: Supplementary file 1 [file Image1.pdf]

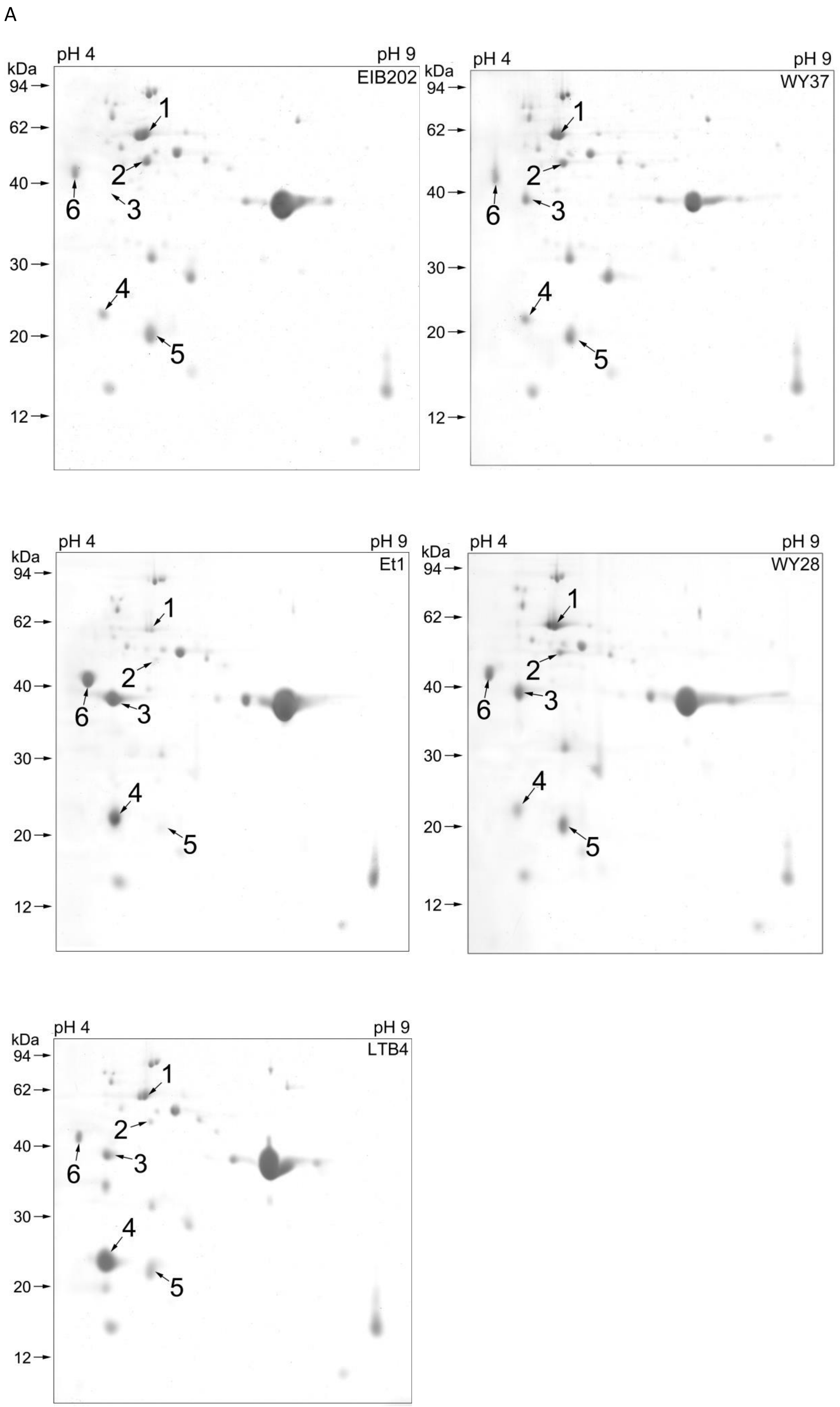
A
pH 4
pH 9
kDa
94
62
40
30
20
12
EIB202
WY37
Et1
WY28
LTB4
1
2
3
4
5
6

B

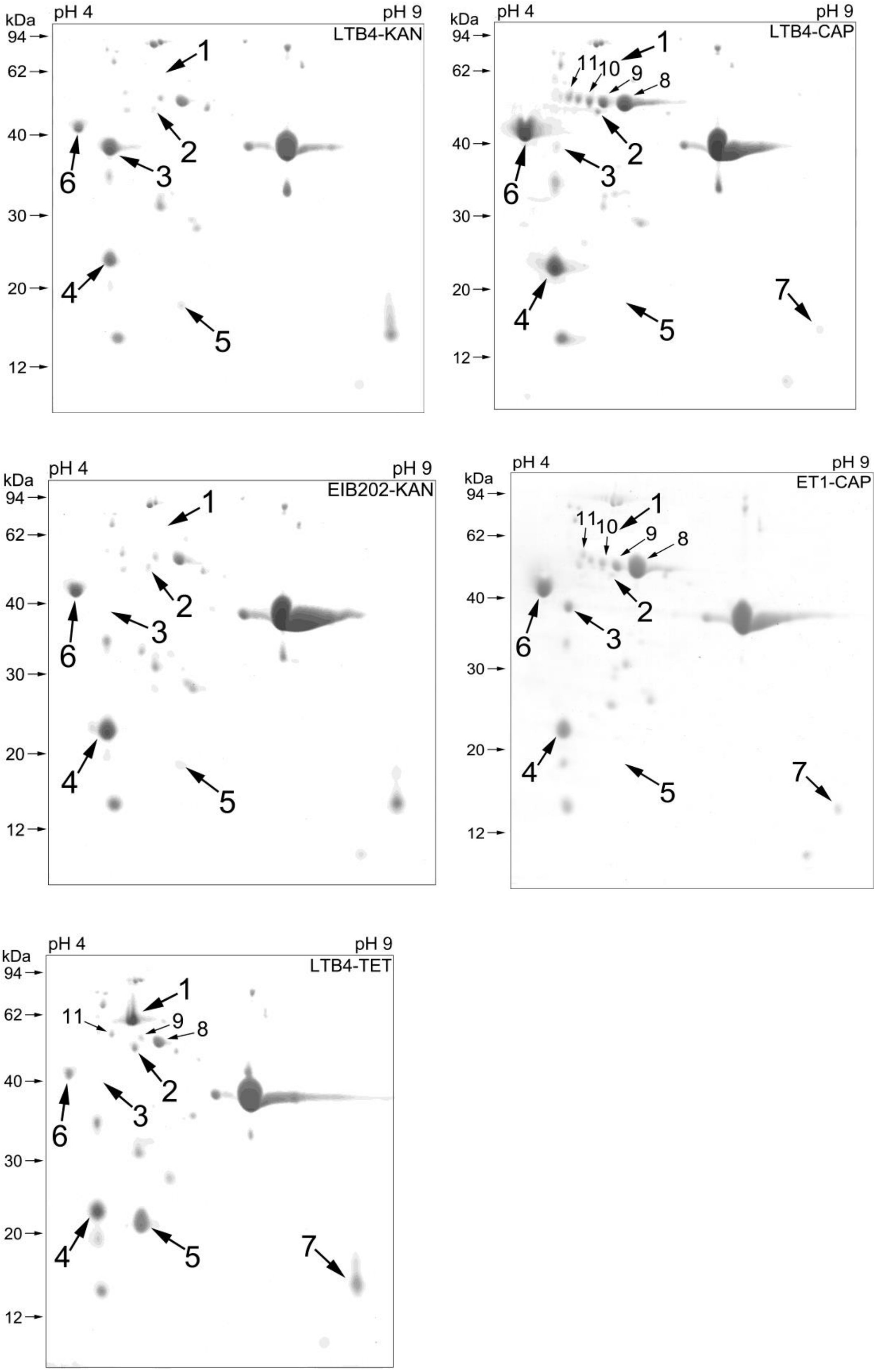

pH 4
pH 9
kDa
94
62
40
30
20
12
LTB4-KAN
LTB4-CAP
EIB202-KAN
ET1-CAP
LTB4-TET
1
2
3
4
5
6
7
8
9
10
11

C

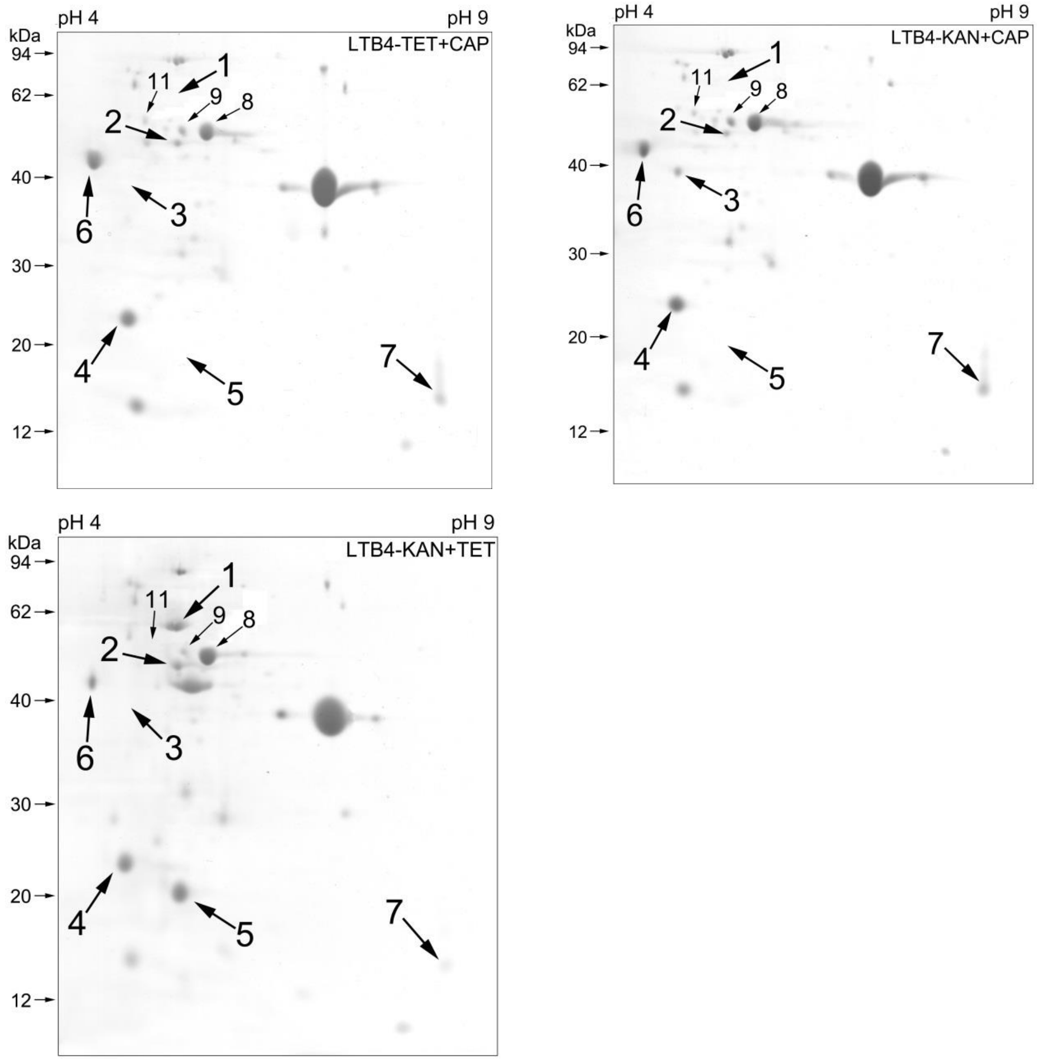

Supplementary Fig. 1 Representative 2-DE maps. A, Five wild strains LTB4, EIB202, WY28, WY37 and Et1. B, Plus one more antibiotic resistance. LTB4 was sub-cultured in medium with KAN, TET or CAP to obtain LTB4-KAN, LTB4-TET, LTB4-CAP, and EIB202 and Et1 were sub-cultured in KAN and CAP to obtain EIB202-KAN and Et1-CAP, respectively. C, Plus two more antibiotic resistance. LTB4 was sub-cultured in medium with two of KAN, TET and CAP to obtain LTB4-KAN/TET, LTB4-KAN/CAP and LTB4-TET/CAP. 1, EvpB; 2, LamB; 3, OmpF2; 4, ETAE_0245; 5, EvpA; 6, ETAE_1826; 7, ETAE_2675; 8, ETAE_0191; 9, ETAE_0191; 10, ETAE_0191; 11, ETAE_0191.
