# Supplementary material for: Outer Membrane Proteins form Specific Patterns in Antibiotic-Resistant Edwardsiella tarda
Source: Front Microbiol. 2017 Feb 2;8:69. doi: 10.3389/fmicb.2017.00069 (PMC5288343; doi:10.3389/fmicb.2017.00069)
Supplement: Supplementary file 2 [file Image2.pdf]

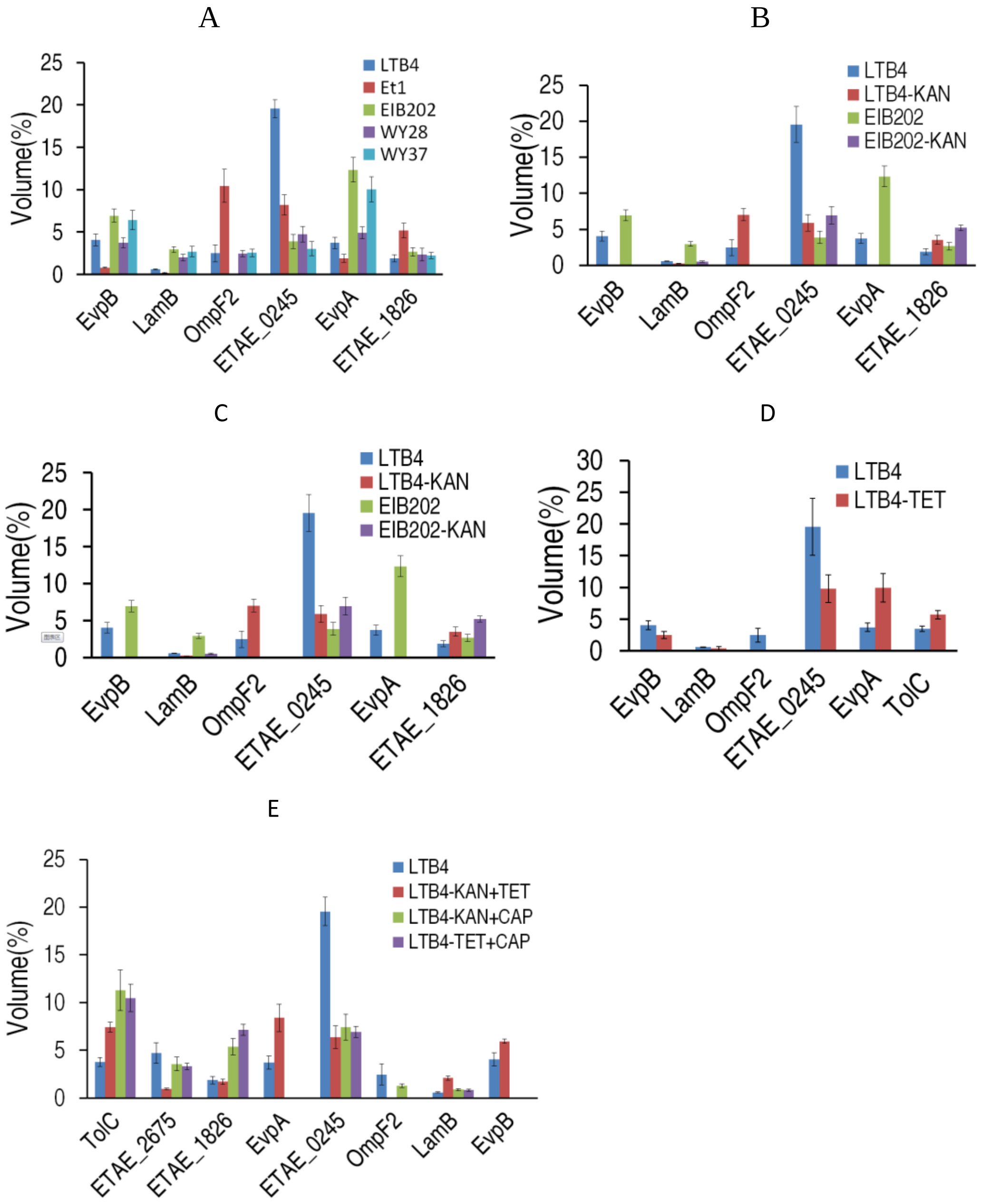

Supplementary Fig. 2 Histogram displays differential abundance of protein spots. They correspond to Fig 1D (A), Fig 2F (B), Fig 2G (C), Fig 2H (D) and Fig 4B (E).
