# Supplementary material for: Outer Membrane Proteins form Specific Patterns in Antibiotic-Resistant Edwardsiella tarda
Source: Front Microbiol. 2017 Feb 2;8:69. doi: 10.3389/fmicb.2017.00069 (PMC5288343; doi:10.3389/fmicb.2017.00069)
Supplement: Supplementary file 3 [file Image3.pdf]

1. ETAE_2430 (EvpB)

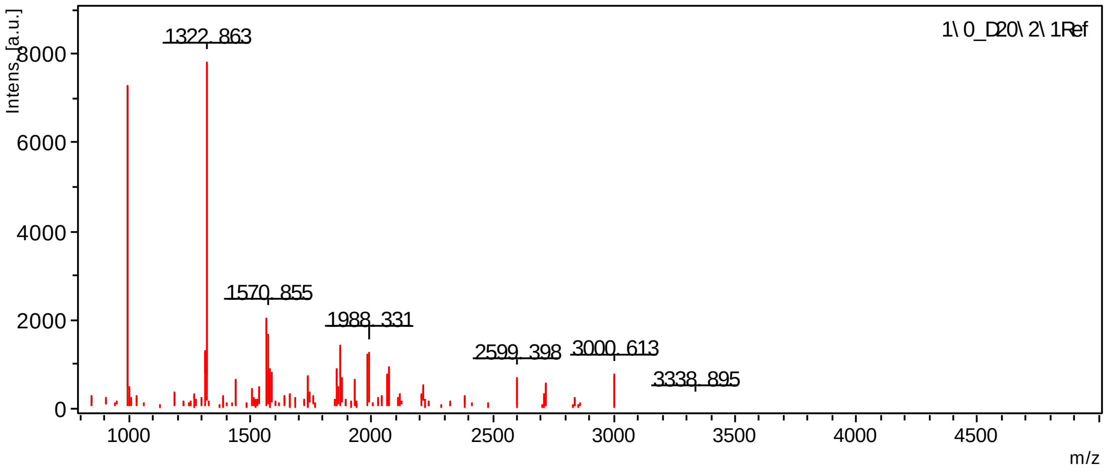

2. ETAE_0214 (LamB)

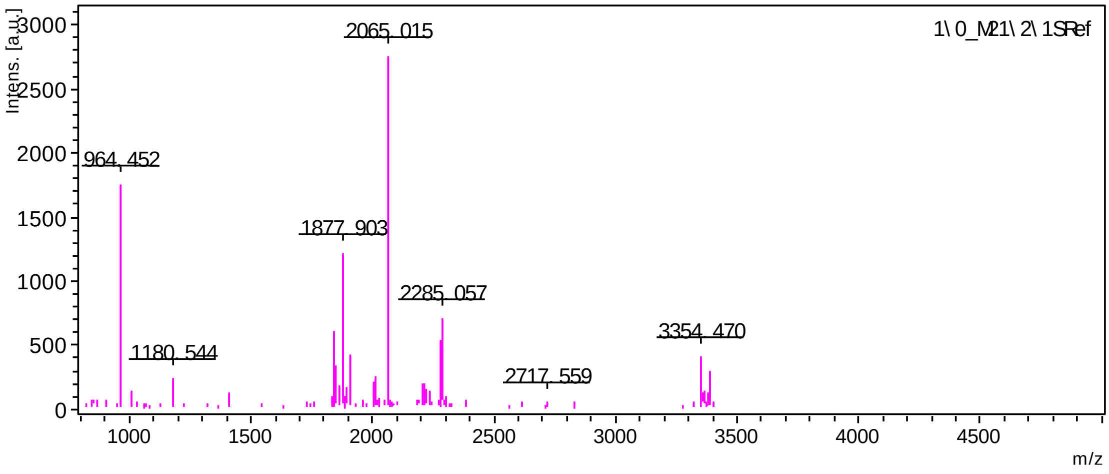

3. ETAE_ 1239 (OmpF2)

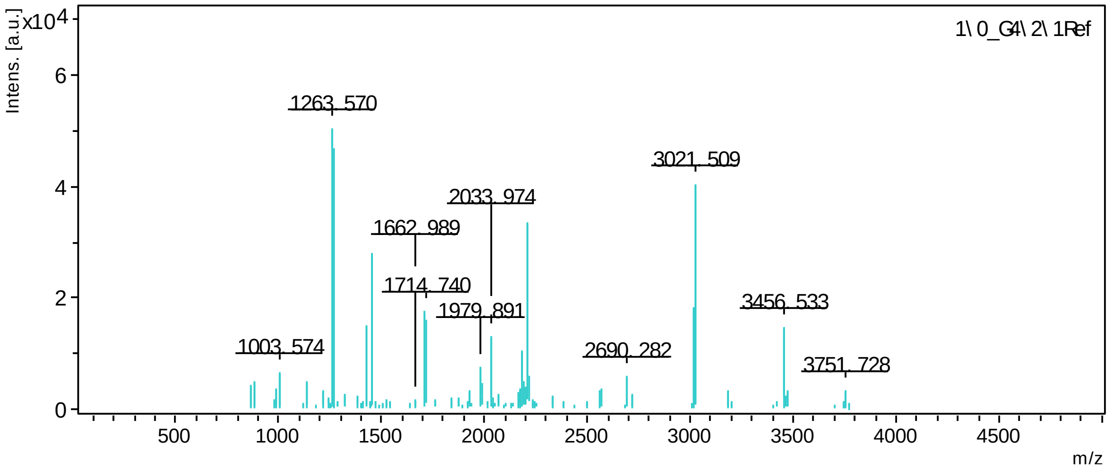

4. ETAE_0245

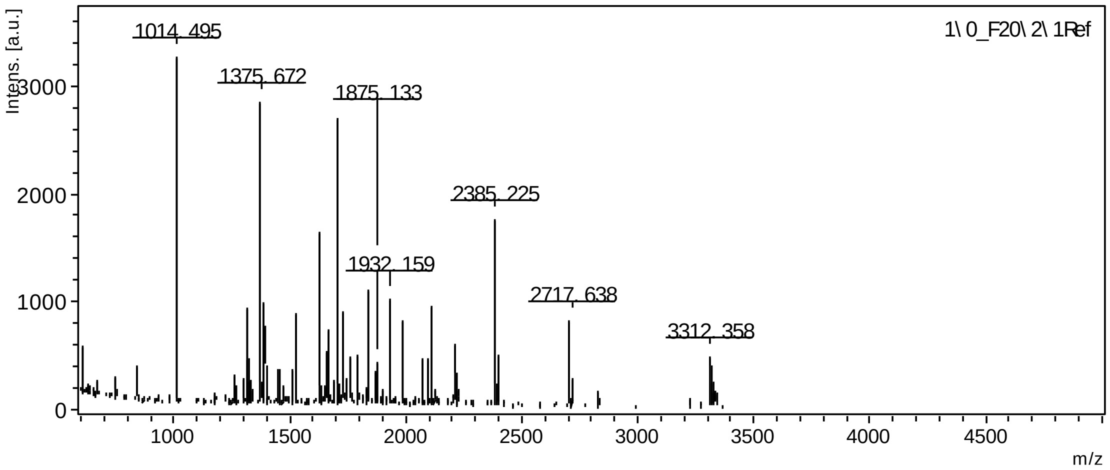

5. EvpA

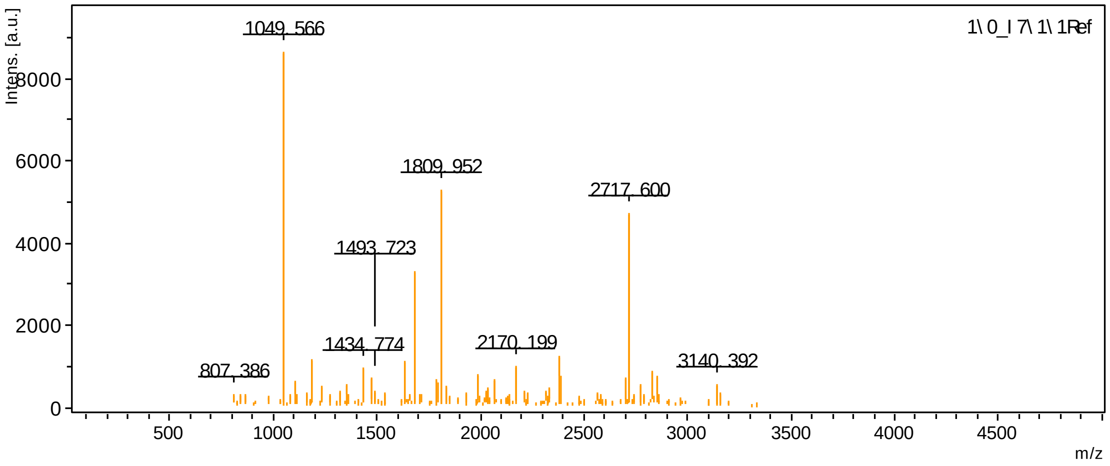

6. ETAE_1826

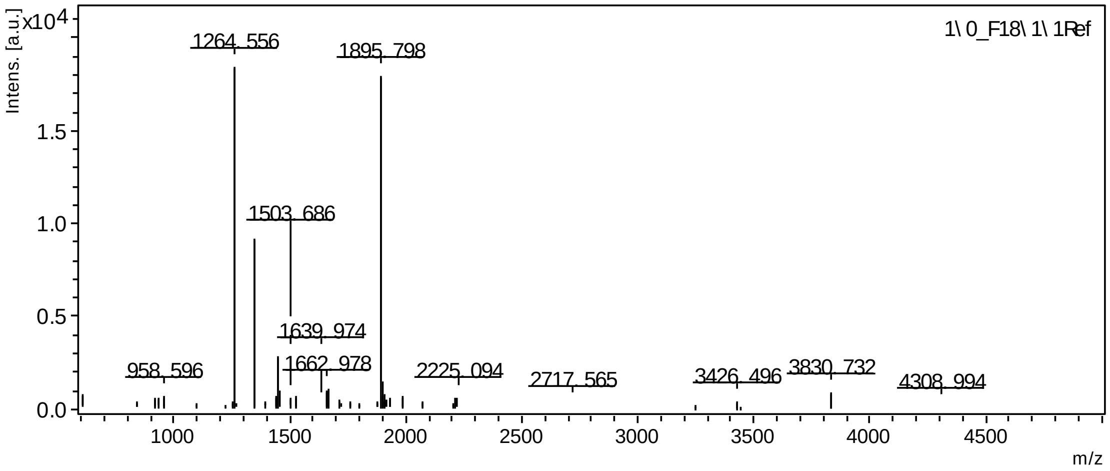

7. ETAE_2675

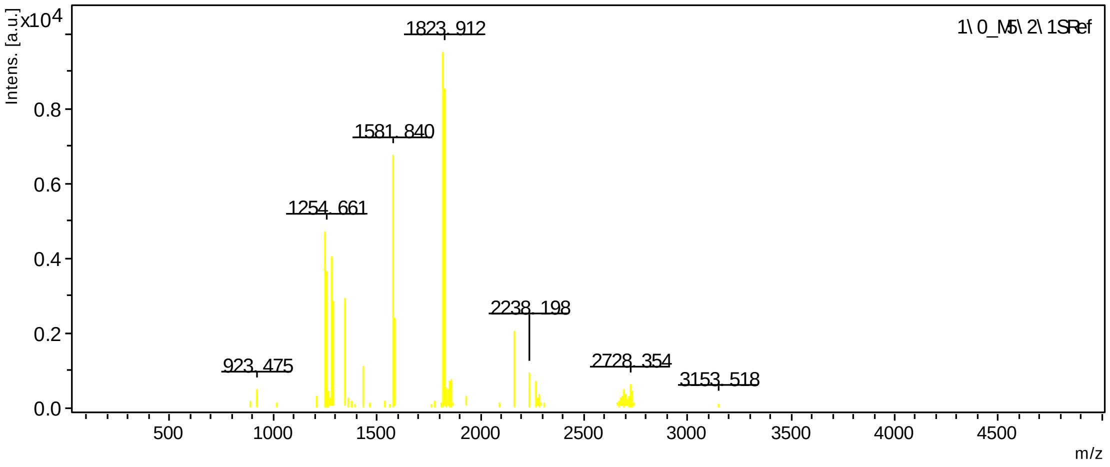

8. ETAE_0191 （TolC）

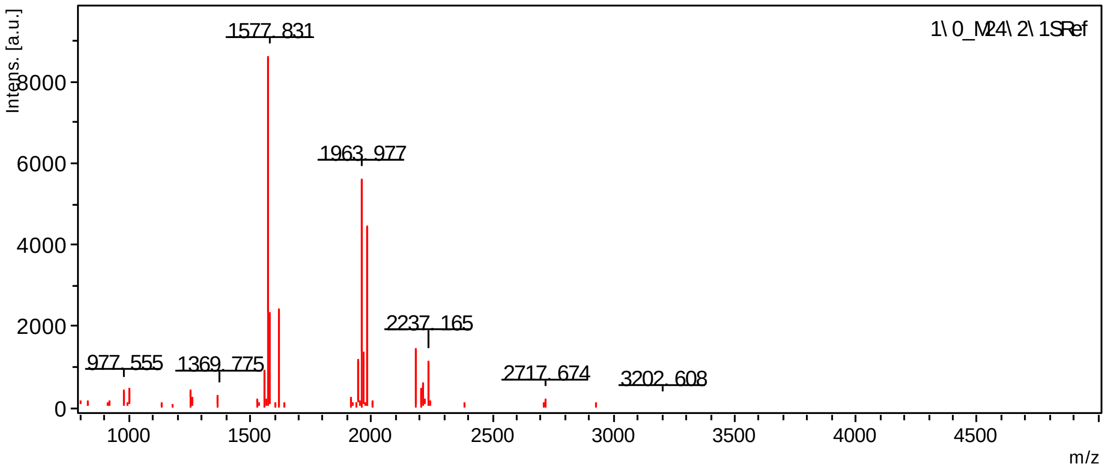

9. ETAE_0191 （TolC）

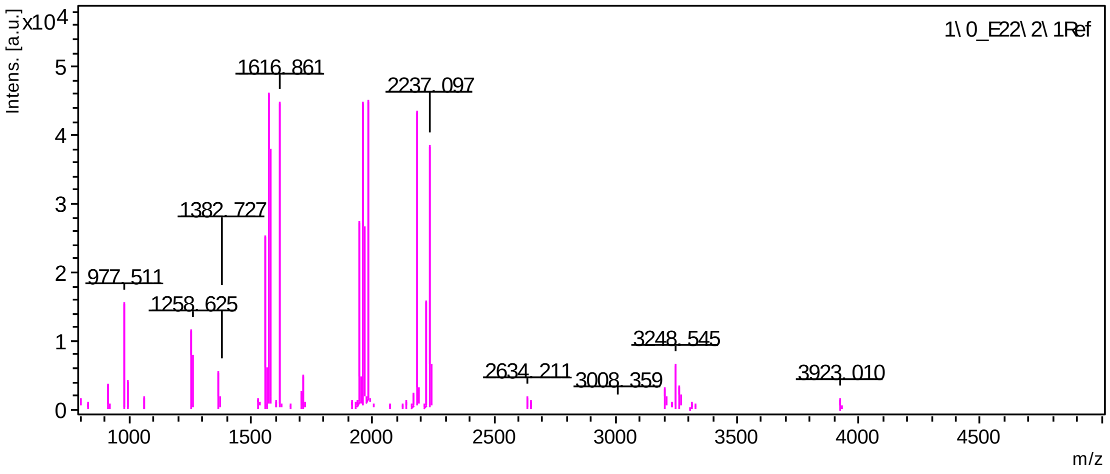

10. ETAE_0191 （TolC）

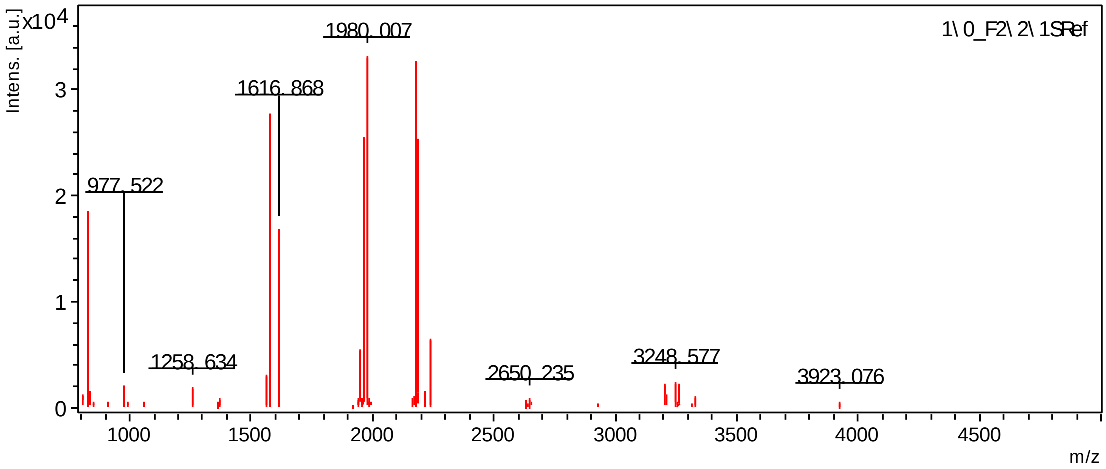

11. ETAE_0191 （TolC）

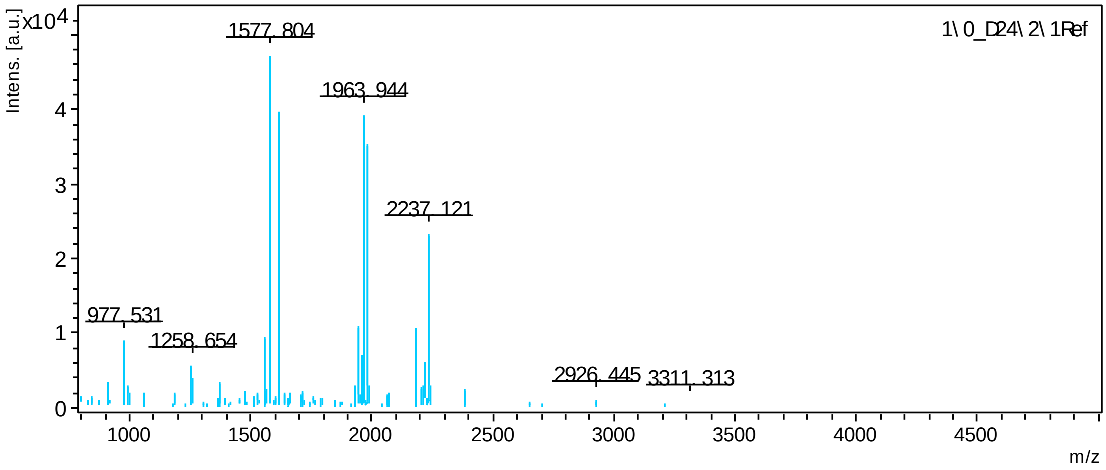

Supplementary Fig. 3 PMF annotated spectra
