# Supplementary material for: Outer Membrane Proteins form Specific Patterns in Antibiotic-Resistant Edwardsiella tarda
Source: Front Microbiol. 2017 Feb 2;8:69. doi: 10.3389/fmicb.2017.00069 (PMC5288343; doi:10.3389/fmicb.2017.00069)
Supplement: Supplementary file 4 [file Table1.DOC]

**Supplementary Table 1 MS analysis of outer membrane proteins of *E. tarda***

| Spot NO. | Accession name | NCBI accession No. | locus_tag | Protein description | Subcellular location | No. of peptides matched | Cover % | Mr/pI | NCBI score |
| --- | --- | --- | --- | --- | --- | --- | --- | --- | --- |
| 1 | D0ZB31_EDWTE | gi|269139775 | ETAE_2430 | type VI secretion system protein EvpB | unknown | 30 | 52 | 54499/5.16 | 307 |
| 2 | D0Z9X1_EDWTE | gi|269137572 | ETAE_0214 | maltoporin | M/OM | 16 | 51 | 47297/5.38 | 155 |
| 3 | D0ZFQ0_EDWTE | gi|269138593 | ETAE_1239 | putative outer membrane porin F protein | M/OM | 15 | 60 | 40058/5.03 | 128 |
| 4 | D0ZA02_EDWTE | gi|269137603 | ETAE_0245 | hypothetical protein ETAE_0245 | OM | 11 | 60 | 22398/5.25 | 103 |
| 5 | Q6EE21_EDWTA | gi|40287636 |  | EvpA(Protein of unknown function ) | unknown | 12 | 70 | 19379/5.29 | 94 |
| 6 | D0Z894_EDWTE | gi|269139173 | ETAE_1826 | putative outer membrane protein (porin) | M/OM | 13 | 59 | 42331/4.68 | 119 |
| 7 | D0ZCH1_EDWTE | gi|269140020 | ETAE_2675 | virulence-related outer membrane protein | M/OM | 14 | 67 | 20476/8.89 | 119 |
| 8 | D0Z9U8_EDWTE | gi|269137549 | ETAE_0191 | outer membrane channel protein | OM | 13 | 37 | 51409/6.64 | 107 |
| 9 | D0Z9U8_EDWTE | gi|269137549 | ETAE_0191 | outer membrane channel protein | OM | 14 | 43 | 51409/6.64 | 109 |
| 10 | D0Z9U8_EDWTE | gi|269137549 | ETAE_0191 | outer membrane channel protein | OM | 16 | 44 | 51409/6.64 | 105 |
| 11 | D0Z9U8_EDWTE | gi|269137549 | ETAE_0191 | outer membrane channel protein | OM | 16 | 42 | 51409/6.64 | 106 |
