# Supplementary material for: Outer Membrane Proteins form Specific Patterns in Antibiotic-Resistant Edwardsiella tarda
Source: Front Microbiol. 2017 Feb 2;8:69. doi: 10.3389/fmicb.2017.00069 (PMC5288343; doi:10.3389/fmicb.2017.00069)
Supplement: Supplementary file 5 [file Table2.DOC]

**Supplementary Table 2 The corresponding amino acid sequences of the protein being reported for each mass-labeled peaks**

| EvpB |  |  |  |  |  |  |  |
| --- | --- | --- | --- | --- | --- | --- | --- |
| **Start** | **End** | **Observed** | **Mr(expt)** | **Mr(calc)** | **ppm** | **M** | **Peptide** |
| 34 | 40 | 818.3904 | 817.3831 | 817.4294 | -56.6 | 1 | R.SDLEKAR.A |
| 71 | 93 | 2511.2671 | 2510.2598 | 2510.3203 | -24.1 | 0 | R.IAQIDALLSAQLSAIMHEPAFQK.L + Oxidation (M) |
| 94 | 115 | 2468.1557 | 2467.1484 | 2467.2608 | -45.5 | 1 | K.LEGSWRGLHYLVHQSETGTGLK.I |
| 100 | 115 | 1739.8499 | 1738.8426 | 1738.9002 | -33.2 | 0 | R.GLHYLVHQSETGTGLK.I |
| 124 | 131 | 977.4852 | 976.4779 | 976.5342 | -57.6 | 1 | R.ADLIRDFK.S |
| 129 | 143 | 1703.7821 | 1702.7749 | 1702.8202 | -26.6 | 1 | R.DFKSAAEFDQSALFK.K |
| 132 | 143 | 1313.5745 | 1312.5672 | 1312.6299 | -47.8 | 0 | K.SAAEFDQSALFK.K |
| 132 | 144 | 1441.678 | 1440.6707 | 1440.7249 | -37.6 | 1 | K.SAAEFDQSALFKK.V |
| 213 | 225 | 1456.6702 | 1455.6629 | 1455.7245 | -42.3 | 1 | R.DLAKGFDTVEYAK.W |
| 217 | 225 | 1029.452 | 1028.4447 | 1028.4815 | -35.7 | 0 | K.GFDTVEYAK.W |
| 217 | 227 | 1343.6192 | 1342.612 | 1342.6557 | -32.6 | 1 | K.GFDTVEYAKWK.S |
| 228 | 236 | 1061.4895 | 1060.4822 | 1060.5261 | -41.4 | 1 | K.SLRQSEDAR.Y |
| 231 | 248 | 2009.0324 | 2008.0251 | 2008.0854 | -30 | 1 | R.QSEDARYIALALPHVLGR.L |
| 237 | 248 | 1322.7402 | 1321.7329 | 1321.787 | -40.9 | 0 | R.YIALALPHVLGR.L |
| 237 | 270 | 3688.8007 | 3687.7935 | 3687.9301 | -37 | 1 | R.YIALALPHVLGRLPYGATTVPVESFNFEENVSGK.E |
| 249 | 270 | 2385.1131 | 2384.1058 | 2384.1536 | -20 | 0 | R.LPYGATTVPVESFNFEENVSGK.E |
| 249 | 274 | 2836.3013 | 2835.2941 | 2835.3715 | -27.3 | 1 | R.LPYGATTVPVESFNFEENVSGKEHGK.Y |
| 271 | 287 | 1962.9389 | 1961.9316 | 1962.0111 | -40.5 | 1 | K.EHGKYLWLNAAYALGTR.L |
| 275 | 287 | 1511.7359 | 1510.7286 | 1510.7932 | -42.8 | 0 | K.YLWLNAAYALGTR.L |
| 303 | 328 | 2599.1939 | 2598.1866 | 2598.245 | -22.5 | 0 | R.GAEGGGLVEGLPAHTFTTDDGEVELK.C |
| 356 | 372 | 1874.8805 | 1873.8732 | 1873.921 | -25.5 | 0 | K.GTDYAAFFSTQSVQKPK.E |
| 356 | 384 | 3168.3677 | 3167.3604 | 3167.4432 | -26.1 | 1 | K.GTDYAAFFSTQSVQKPKEYDSDSANANAR.I |
| 373 | 384 | 1312.4793 | 1311.472 | 1311.5327 | -46.3 | 0 | K.EYDSDSANANAR.I |
| 398 | 407 | 1267.6104 | 1266.6031 | 1266.6543 | -40.4 | 1 | R.FAHYLKSMVR.D + Oxidation (M) |
| 408 | 416 | 1056.4651 | 1055.4578 | 1055.507 | -46.6 | 1 | R.DKIGSFMSR.S + Oxidation (M) |
| 410 | 416 | 813.3454 | 812.3381 | 812.3851 | -57.8 | 0 | K.IGSFMSR.S + Oxidation (M) |
| 445 | 451 | 904.4524 | 903.4451 | 903.4926 | -52.5 | 1 | K.YPLREAR.I |
| 452 | 465 | 1535.7599 | 1534.7526 | 1534.8031 | -32.9 | 0 | R.IDVSDIPGKPGFYK.A |
| 452 | 484 | 3630.8415 | 3629.8342 | 3629.961 | -34.9 | 1 | R.IDVSDIPGKPGFYKAVAYLKPHFQLEGLTASLR.L |
| 466 | 484 | 2114.1097 | 2113.1024 | 2113.1684 | -31.2 | 0 | K.AVAYLKPHFQLEGLTASLR.L |

| LamB |  |  |  |  |  |  |  |
| --- | --- | --- | --- | --- | --- | --- | --- |
| **Start** | **End** | **Observed** | **Mr(expt)** | **Mr(calc)** | **ppm** | **M** | **Peptide** |
| 56 | 69 | 1731.748 | 1730.7407 | 1730.7933 | -30.4 | 1 | K.YRLGNECDTYAELK.L |
| 58 | 69 | 1412.6028 | 1411.5955 | 1411.6289 | -23.7 | 0 | R.LGNECDTYAELK.L |
| 70 | 76 | 873.4614 | 872.4541 | 872.4756 | -24.6 | 0 | K.LGQELWK.S |
| 77 | 106 | 3354.4063 | 3353.399 | 3353.4749 | -22.6 | 0 | K.SGDQSFYLDTNVAYGIDQANDWESTSPAFR.E |
| 114 | 128 | 1642.8467 | 1641.8394 | 1641.8879 | -29.5 | 0 | K.NVITWLPGSTLWAGK.R |
| 170 | 187 | 2010.8105 | 2009.8032 | 2009.8603 | -28.4 | 0 | R.NTESGGSYSYFVNQSNEK.Y |
| 191 | 200 | 1180.5263 | 1179.519 | 1179.552 | -28 | 0 | R.NTANDVYDIR.L |
| 201 | 220 | 2064.9822 | 2063.9749 | 2064.0276 | -25.5 | 0 | R.LAGLQTNPGGSLELGFDYGR.A |
| 221 | 235 | 1542.7142 | 1541.707 | 1541.7474 | -26.2 | 0 | R.ANLDDGYHLAPGATK.D |
| 236 | 254 | 2188.9274 | 2187.9202 | 2187.9684 | -22 | 0 | K.DGYMFTAEHTQSLWGGFNK.F |
| 236 | 254 | 2204.9115 | 2203.9042 | 2203.9633 | -26.8 | 0 | K.DGYMFTAEHTQSLWGGFNK.F + Oxidation (M) |
| 255 | 286 | 3363.4605 | 3362.4532 | 3362.5334 | -23.9 | 0 | K.FVVQYATDAMSNTNNAGHSSGSSVNNDGHLIR.V |
| 255 | 286 | 3379.4457 | 3378.4384 | 3378.5284 | -26.6 | 0 | K.FVVQYATDAMSNTNNAGHSSGSSVNNDGHLIR.V + Oxidation (M) |
| 331 | 347 | 1959.8629 | 1958.8556 | 1958.9084 | -27 | 0 | K.WTPTMSTLFEAGYDNVK.S |
| 331 | 347 | 1975.8518 | 1974.8445 | 1974.9033 | -29.8 | 0 | K.WTPTMSTLFEAGYDNVK.S + Oxidation (M) |
| 389 | 408 | 2285.0365 | 2284.0293 | 2284.0832 | -23.6 | 1 | K.NNWNTDKDGIQVNTQPGDIR.A |

| OmpF2 |  |  |  |  |  |  |  |
| --- | --- | --- | --- | --- | --- | --- | --- |
| **Start** | **End** | **Observed** | **Mr(expt)** | **Mr(calc)** | **ppm** | **M** | **Peptide** |
| 45 | 59 | 1714.7706 | 1713.7633 | 1713.7231 | 23.5 | 1 | K.FTSDKSDDGDHTYAR.F |
| 60 | 83 | 2690.3495 | 2689.3423 | 2689.266 | 28.4 | 1 | R.FGFKGETQINSELTGYGQWEAEAK.A |
| 105 | 115 | 1263.5993 | 1262.592 | 1262.5568 | 27.9 | 0 | K.FADYGSLDYGR.N |
| 116 | 148 | 3751.8314 | 3750.8241 | 3750.6825 | 37.8 | 0 | R.NYGVVYDVEAWTDVLPVFGGDSYTYTDNFMTGR.T |
| 149 | 156 | 881.4592 | 880.4519 | 880.4403 | 13.2 | 0 | R.TNGVATYR.N |
| 157 | 176 | 2186.129 | 2185.1217 | 2185.0626 | 27 | 0 | R.NNGFFGLVDGLNMALQYQGK.N |
| 227 | 246 | 1987.1908 | 1986.1835 | 1986.0534 | 65.5 | 1 | K.NAGGLIASGDKAQVWTTGLK.Y |
| 238 | 246 | 1003.586 | 1002.5787 | 1002.5498 | 28.8 | 0 | K.AQVWTTGLK.Y |
| 247 | 275 | 3184.5363 | 3183.529 | 3183.4165 | 35.3 | 0 | K.YDANNVYVAAMYAETLNMTPYGDDGIANK.T |
| 276 | 301 | 3021.5968 | 3020.5895 | 3020.5032 | 28.6 | 0 | K.TQNFEAVAQYQFDFGLRPSIAYLQSK.G |
| 276 | 303 | 3206.4721 | 3205.4648 | 3205.6196 | -48.3 | 1 | K.TQNFEAVAQYQFDFGLRPSIAYLQSKGK.Q |
| 318 | 329 | 1453.7491 | 1452.7418 | 1452.6925 | 33.9 | 0 | K.YVDLGATYYFNK.N |
| 330 | 338 | 1120.5286 | 1119.5213 | 1119.4906 | 27.4 | 0 | K.NMSTYVDYK.I |
| 330 | 338 | 1136.4902 | 1135.4829 | 1135.4856 | -2.35 | 0 | K.NMSTYVDYK.I + Oxidation (M) |
| 339 | 350 | 1426.7269 | 1425.7196 | 1425.6776 | 29.5 | 0 | K.INLLDGNDDFYK.T |

| ETAE_0245 |  |  |  |  |  |  |  |
| --- | --- | --- | --- | --- | --- | --- | --- |
| **Start** | **End** | **Observed** | **Mr(expt)** | **Mr(calc)** | **ppm** | **M** | **Peptide** |
| 36 | 47 | 1375.6656 | 1374.6584 | 1374.6602 | -1.31 | 0 | K.MGISAEHLFDQK.D |
| 36 | 47 | 1391.6604 | 1390.6531 | 1390.6551 | -1.44 | 0 | K.MGISAEHLFDQK.D + Oxidation (M) |
| 60 | 81 | 2385.1691 | 2384.1618 | 2384.1913 | -12.4 | 0 | K.GVGNAGLVFGYNFENQFSLPVR.A |
| 82 | 89 | 1014.4847 | 1013.4774 | 1013.4818 | -4.35 | 0 | R.AELDYTFR.A |
| 108 | 122 | 1727.8937 | 1726.8865 | 1726.8964 | -5.74 | 0 | R.LGLQTLMVNGYYDIK.T |
| 108 | 122 | 1743.8877 | 1742.8804 | 1742.8913 | -6.25 | 0 | R.LGLQTLMVNGYYDIK.T + Oxidation (M) |
| 123 | 143 | 2113.0791 | 2112.0718 | 2112.0892 | -8.23 | 0 | K.TGTPFTPYVGAGIGYANVSLK.N |
| 144 | 173 | 3315.4534 | 3314.4462 | 3314.4647 | -5.59 | 0 | K.NDMNGDNVQSNSNNFAWSVGTGVIYNVNER.L |
| 144 | 173 | 3331.4378 | 3330.4306 | 3330.4596 | -8.71 | 0 | K.NDMNGDNVQSNSNNFAWSVGTGVIYNVNER.L + Oxidation (M) |
| 174 | 181 | 936.4935 | 935.4863 | 935.4964 | -10.8 | 0 | R.LDLDLGYK.Y |
| 197 | 210 | 1628.7915 | 1627.7843 | 1627.7883 | -2.45 | 0 | K.VTTHDVTLGVNYYF. |

| EvpA |  |  |  |  |  |  |  |
| --- | --- | --- | --- | --- | --- | --- | --- |
| **Start** | **End** | **Observed** | **Mr(expt)** | **Mr(calc)** | **ppm** | **M** | **Peptide** |
| 18 | 33 | 1854.8623 | 1853.855 | 1853.8717 | -9.01 | 0 | R.VQITYDVEIGDAQEMK.E + Oxidation (M) |
| 34 | 55 | 2316.1895 | 2315.1822 | 2315.1872 | -2.12 | 0 | K.ELPFVMGVLGDYSGQPATPLPK.L |
| 34 | 55 | 2332.1922 | 2331.1849 | 2331.1821 | 1.2 | 0 | K.ELPFVMGVLGDYSGQPATPLPK.L + Oxidation (M) |
| 60 | 66 | 864.501 | 863.4937 | 863.4865 | 8.36 | 1 | R.KFVSIDR.D |
| 61 | 74 | 1681.8558 | 1680.8485 | 1680.8471 | 0.8 | 1 | K.FVSIDRDNFNDVIK.G |
| 75 | 83 | 1049.5655 | 1048.5582 | 1048.5567 | 1.51 | 0 | K.GVHPHLSFR.T |
| 84 | 111 | 3140.3923 | 3139.385 | 3139.4041 | -6.06 | 0 | R.TENTLSGDDSQLSVDLHFQSMADFTPER.V |
| 84 | 111 | 3156.4086 | 3155.4013 | 3155.399 | 0.73 | 0 | R.TENTLSGDDSQLSVDLHFQSMADFTPER.V + Oxidation (M) |
| 112 | 120 | 982.5662 | 981.5589 | 981.5607 | -1.82 | 0 | R.VAAQVEPLR.K |
| 112 | 121 | 1110.6645 | 1109.6572 | 1109.6557 | 1.36 | 1 | R.VAAQVEPLRK.L |
| 142 | 155 | 1543.8337 | 1542.8264 | 1542.8141 | 7.97 | 0 | R.LGEVLQGIIEDTEK.L |
| 142 | 161 | 2170.1995 | 2169.1922 | 2169.1892 | 1.37 | 1 | R.LGEVLQGIIEDTEKLQSLGK.E |

| ETAE_1826 |  |  |  |  |  |  |  |
| --- | --- | --- | --- | --- | --- | --- | --- |
| **Start** | **End** | **Observed** | **Mr(expt)** | **Mr(calc)** | **ppm** | **M** | **Peptide** |
| 51 | 58 | 939.4152 | 938.408 | 938.4094 | -1.51 | 0 | K.DGDQSYVR.F |
| 63 | 96 | 3830.7321 | 3829.7248 | 3829.7204 | 1.14 | 0 | K.GETQINDQLTGYGQWEAQANVNQPESNSSNFFTR.L |
| 104 | 114 | 1264.5563 | 1263.549 | 1263.552 | -2.35 | 0 | K.YGNYGSIDYGR.N |
| 115 | 147 | 3725.6182 | 3724.6109 | 3724.6628 | -13.9 | 0 | R.NYGVLYDIEGWTDVLPEFGGDTSAQTDNYMAQR.A |
| 148 | 155 | 922.475 | 921.4677 | 921.4668 | 0.97 | 0 | R.ANNLATYR.N |
| 156 | 175 | 2203.0643 | 2202.0571 | 2202.0746 | -7.96 | 0 | R.NNGFFGLVDGLDFALQYQGK.N |
| 184 | 201 | 1895.798 | 1894.7907 | 1894.8082 | -9.22 | 0 | K.DADGSYSWDGAPLSNNAR.E |
| 240 | 254 | 1503.6864 | 1502.6792 | 1502.6862 | -4.68 | 0 | K.WSGNQANSNVAGGNK.A |
| 255 | 263 | 917.4871 | 916.4798 | 916.4767 | 3.47 | 0 | K.AQSWGAGLK.Y |
| 264 | 292 | 3245.455 | 3244.4477 | 3244.4693 | -6.63 | 0 | K.YDANNIYLATMYTETQNMTPFGSTGIANK.A + Oxidation (M) |
| 295 | 318 | 2792.3651 | 2791.3578 | 2791.3969 | -14 | 0 | K.NFEAVAQYQFDFGLRPSIAYLQSK.A |
| 338 | 349 | 1453.6924 | 1452.6851 | 1452.6925 | -5.1 | 0 | K.YVDLGATYYFNK.N |
| 359 | 369 | 1349.6627 | 1348.6554 | 1348.6623 | -5.08 | 0 | K.INLLDENDFTR.A |

| ETAE_2675 |  |  |  |  |  |  |  |
| --- | --- | --- | --- | --- | --- | --- | --- |
| **Start** | **End** | **Observed** | **Mr(expt)** | **Mr(calc)** | **ppm** | **M** | **Peptide** |
| 49 | 70 | 2665.0896 | 2664.0823 | 2664.1876 | -39.5 | 1 | K.YRYEWDSPLSLMTSFTYMSGSK.A + Oxidation (M) |
| 49 | 70 | 2681.0898 | 2680.0825 | 2680.1825 | -37.3 | 1 | K.YRYEWDSPLSLMTSFTYMSGSK.A + 2 Oxidation (M) |
| 51 | 70 | 2361.9429 | 2360.9356 | 2361.0181 | -34.9 | 0 | R.YEWDSPLSLMTSFTYMSGSK.A + 2 Oxidation (M) |
| 71 | 82 | 1346.5628 | 1345.5556 | 1345.615 | -44.2 | 0 | K.AFSESTGLEYSR.G |
| 89 | 99 | 1254.5695 | 1253.5622 | 1253.6193 | -45.5 | 0 | K.YYSLAAGPAWR.I |
| 100 | 116 | 1790.8795 | 1789.8722 | 1789.9574 | -47.6 | 0 | R.INDSVSVYGLLGVNVNK.T |
| 117 | 124 | 891.4152 | 890.4079 | 890.461 | -59.6 | 0 | K.TTVSAAWR.D |
| 117 | 140 | 2696.1452 | 2695.1379 | 2695.2991 | -59.8 | 1 | K.TTVSAAWRDVWVGGYEEGSLQQTR.S |
| 125 | 140 | 1823.7811 | 1822.7738 | 1822.8486 | -41 | 0 | R.DVWVGGYEEGSLQQTR.S |
| 141 | 169 | 3099.3756 | 3098.3684 | 3098.5131 | -46.7 | 1 | R.SKGSLMYGAGLQINPLANWSIDVGYEGSR.V + Oxidation (M) |
| 143 | 169 | 2868.2781 | 2867.2709 | 2867.3912 | -42 | 0 | K.GSLMYGAGLQINPLANWSIDVGYEGSR.V |
| 143 | 169 | 2884.2485 | 2883.2412 | 2883.3861 | -50.3 | 0 | K.GSLMYGAGLQINPLANWSIDVGYEGSR.V + Oxidation (M) |
| 177 | 189 | 1433.6592 | 1432.6519 | 1432.7211 | -48.3 | 0 | K.HSINGFNLGVGYR.F |
| 177 | 190 | 1580.7329 | 1579.7256 | 1579.7896 | -40.5 | 1 | K.HSINGFNLGVGYRF.- |

| TolC 1 |  |  |  |  |  |  |  |
| --- | --- | --- | --- | --- | --- | --- | --- |
| **Start** | **End** | **Observed** | **Mr(expt)** | **Mr(calc)** | **ppm** | **M** | **Peptide** |
| 141 | 152 | 1366.6899 | 1365.6826 | 1365.6776 | 3.66 | 0 | R.AIDQLTYTEANK.Q |
| 141 | 165 | 2926.4653 | 2925.4581 | 2925.4468 | 3.85 | 1 | R.AIDQLTYTEANKQAIYNQLDQTTQR.F |
| 166 | 180 | 1616.8932 | 1615.8859 | 1615.8682 | 11 | 0 | R.FNVGLVAITDVQNAR.A |
| 195 | 205 | 1258.6515 | 1257.6442 | 1257.6313 | 10.3 | 0 | R.NNLDNSLEALR.Q |
| 206 | 222 | 1980.0377 | 1979.0305 | 1979.0112 | 9.73 | 0 | R.QITGQYYPQLSALNIDR.F |
| 233 | 241 | 1057.6071 | 1056.5999 | 1056.5927 | 6.78 | 1 | K.ALLQEAEKR.N |
| 242 | 249 | 914.5507 | 913.5434 | 913.5345 | 9.79 | 0 | R.NLQLLSAR.L |
| 250 | 256 | 802.4329 | 801.4256 | 801.4344 | -11.1 | 0 | R.LSQDLAR.Q |
| 261 | 291 | 3327.5561 | 3326.5489 | 3326.5249 | 7.22 | 0 | K.YAETGYMPTLNLTASTGVSNTDYNSLSNAQK.A + Oxidation (M) |
| 345 | 351 | 801.464 | 800.4567 | 800.4505 | 7.82 | 0 | R.SVVQNVR.S |
| 369 | 390 | 2237.1343 | 2236.127 | 2236.1084 | 8.32 | 0 | K.QAVVSAQSSLDATQAGYQVGTR.T |
| 391 | 406 | 1706.9371 | 1705.9298 | 1705.925 | 2.82 | 0 | R.TIVDVLNATTALYNAK.Q |
| 414 | 424 | 1369.7544 | 1368.7472 | 1368.7289 | 13.4 | 0 | R.YDYLISQLNIK.Y |

| TolC 2 |  |  |  |  |  |  |  |
| --- | --- | --- | --- | --- | --- | --- | --- |
| **Start** | **End** | **Observed** | **Mr(expt)** | **Mr(calc)** | **ppm** | **M** | **Peptide** |
| 112 | 140 | 3248.5565 | 3247.5492 | 3247.5819 | -10.1 | 0 | K.QAGIQDVSYQSSEQTLMLNTATAYFNVLR.A |
| 141 | 165 | 2926.4451 | 2925.4378 | 2925.4468 | -3.08 | 1 | R.AIDQLTYTEANKQAIYNQLDQTTQR.F |
| 166 | 180 | 1616.8911 | 1615.8839 | 1615.8682 | 9.68 | 0 | R.FNVGLVAITDVQNAR.A |
| 195 | 205 | 1258.6538 | 1257.6465 | 1257.6313 | 12.1 | 0 | R.NNLDNSLEALR.Q |
| 206 | 222 | 1980.0305 | 1979.0233 | 1979.0112 | 6.08 | 0 | R.QITGQYYPQLSALNIDR.F |
| 233 | 241 | 1057.6147 | 1056.6075 | 1056.5927 | 14 | 1 | K.ALLQEAEKR.N |
| 242 | 249 | 914.5545 | 913.5472 | 913.5345 | 14 | 0 | R.NLQLLSAR.L |
| 250 | 256 | 802.4537 | 801.4464 | 801.4344 | 15 | 0 | R.LSQDLAR.Q |
| 261 | 291 | 3311.3127 | 3310.3055 | 3310.5299 | -67.8 | 0 | K.YAETGYMPTLNLTASTGVSNTDYNSLSNAQK.A |
| 261 | 291 | 3327.5336 | 3326.5263 | 3326.5249 | 0.43 | 0 | K.YAETGYMPTLNLTASTGVSNTDYNSLSNAQK.A + Oxidation (M) |
| 345 | 351 | 801.4631 | 800.4559 | 800.4505 | 6.74 | 0 | R.SVVQNVR.S |
| 369 | 390 | 2237.1205 | 2236.1132 | 2236.1084 | 2.16 | 0 | K.QAVVSAQSSLDATQAGYQVGTR.T |
| 391 | 406 | 1706.93 | 1705.9228 | 1705.925 | -1.33 | 0 | R.TIVDVLNATTALYNAK.Q |
| 414 | 424 | 1369.7554 | 1368.7481 | 1368.7289 | 14 | 0 | R.YDYLISQLNIK.Y |

| TolC 3 |  |  |  |  |  |  |  |
| --- | --- | --- | --- | --- | --- | --- | --- |
| **Start** | **End** | **Observed** | **Mr(expt)** | **Mr(calc)** | **ppm** | **M** | **Peptide** |
| 34 | 40 | 830.3965 | 829.3892 | 829.393 | -4.54 | 0 | K.ESNPDLR.Q |
| 58 | 77 | 2183.1011 | 2182.0938 | 2182.1059 | -5.51 | 0 | R.SPLLPQLGLGADYTYTNGFR.D |
| 112 | 140 | 3248.5332 | 3247.5259 | 3247.5819 | -17.2 | 0 | K.QAGIQDVSYQSSEQTLMLNTATAYFNVLR.A |
| 166 | 180 | 1616.8599 | 1615.8526 | 1615.8682 | -9.66 | 0 | R.FNVGLVAITDVQNAR.A |
| 195 | 205 | 1258.6235 | 1257.6162 | 1257.6313 | -12 | 0 | R.NNLDNSLEALR.Q |
| 206 | 222 | 1979.9993 | 1978.9921 | 1979.0112 | -9.67 | 0 | R.QITGQYYPQLSALNIDR.F |
| 233 | 241 | 1057.5892 | 1056.582 | 1056.5927 | -10.2 | 1 | K.ALLQEAEKR.N |
| 242 | 249 | 914.5344 | 913.5271 | 913.5345 | -8.06 | 0 | R.NLQLLSAR.L |
| 250 | 256 | 802.4341 | 801.4268 | 801.4344 | -9.51 | 0 | R.LSQDLAR.Q |
| 261 | 291 | 3311.505 | 3310.4977 | 3310.5299 | -9.74 | 0 | K.YAETGYMPTLNLTASTGVSNTDYNSLSNAQK.A |
| 261 | 291 | 3327.5045 | 3326.4972 | 3326.5249 | -8.3 | 0 | K.YAETGYMPTLNLTASTGVSNTDYNSLSNAQK.A + Oxidation (M) |
| 345 | 351 | 801.4488 | 800.4415 | 800.4505 | -11.2 | 0 | R.SVVQNVR.S |
| 369 | 390 | 2237.0932 | 2236.0859 | 2236.1084 | -10.1 | 0 | K.QAVVSAQSSLDATQAGYQVGTR.T |
| 369 | 406 | 3924.7069 | 3923.6997 | 3924.0229 | -82.4 | 1 | K.QAVVSAQSSLDATQAGYQVGTRTIVDVLNATTALYNAK.Q |
| 391 | 406 | 1706.9249 | 1705.9177 | 1705.925 | -4.33 | 0 | R.TIVDVLNATTALYNAK.Q |
| 414 | 424 | 1369.7251 | 1368.7179 | 1368.7289 | -8.05 | 0 | R.YDYLISQLNIK.Y |

| TolC 4 |  |  |  |  |  |  |  |
| --- | --- | --- | --- | --- | --- | --- | --- |
| **Start** | **End** | **Observed** | **Mr(expt)** | **Mr(calc)** | **ppm** | **M** | **Peptide** |
| 34 | 40 | 830.4046 | 829.3973 | 829.393 | 5.19 | 0 | K.ESNPDLR.Q |
| 112 | 140 | 3248.5446 | 3247.5373 | 3247.5819 | -13.7 | 0 | K.QAGIQDVSYQSSEQTLMLNTATAYFNVLR.A |
| 141 | 152 | 1366.6685 | 1365.6613 | 1365.6776 | -12 | 0 | R.AIDQLTYTEANK.Q |
| 166 | 180 | 1616.8609 | 1615.8536 | 1615.8682 | -9.06 | 0 | R.FNVGLVAITDVQNAR.A |
| 195 | 205 | 1258.6249 | 1257.6177 | 1257.6313 | -10.8 | 0 | R.NNLDNSLEALR.Q |
| 206 | 222 | 1979.9987 | 1978.9914 | 1979.0112 | -10 | 0 | R.QITGQYYPQLSALNIDR.F |
| 233 | 241 | 1057.5936 | 1056.5864 | 1056.5927 | -6.01 | 1 | K.ALLQEAEKR.N |
| 242 | 249 | 914.5396 | 913.5323 | 913.5345 | -2.39 | 0 | R.NLQLLSAR.L |
| 250 | 256 | 802.4339 | 801.4266 | 801.4344 | -9.79 | 0 | R.LSQDLAR.Q |
| 261 | 291 | 3311.5143 | 3310.507 | 3310.5299 | -6.93 | 0 | K.YAETGYMPTLNLTASTGVSNTDYNSLSNAQK.A |
| 261 | 291 | 3327.5047 | 3326.4974 | 3326.5249 | -8.24 | 0 | K.YAETGYMPTLNLTASTGVSNTDYNSLSNAQK.A + Oxidation (M) |
| 345 | 351 | 801.4538 | 800.4465 | 800.4505 | -4.97 | 0 | R.SVVQNVR.S |
| 369 | 390 | 2237.0968 | 2236.0896 | 2236.1084 | -8.42 | 0 | K.QAVVSAQSSLDATQAGYQVGTR.T |
| 369 | 406 | 3925.0711 | 3924.0638 | 3924.0229 | 10.4 | 1 | K.QAVVSAQSSLDATQAGYQVGTRTIVDVLNATTALYNAK.Q |
| 391 | 406 | 1706.9229 | 1705.9156 | 1705.925 | -5.51 | 0 | R.TIVDVLNATTALYNAK.Q |
| 414 | 424 | 1369.7255 | 1368.7182 | 1368.7289 | -7.8 | 0 | R.YDYLISQLNIK.Y |
